# Mitochondrial SSBP1 protects cells from proteotoxic stresses by potentiating stress-induced HSF1 transcriptional activity

**DOI:** 10.1038/ncomms7580

**Published:** 2015-03-12

**Authors:** Ke Tan, Mitsuaki Fujimoto, Ryosuke Takii, Eiichi Takaki, Naoki Hayashida, Akira Nakai

**Affiliations:** 1 Department of Biochemistry and Molecular Biology, Yamaguchi University School of Medicine, Minami-Kogushi 1-1-1, Ube 755-8505, Japan

## Abstract

Heat-shock response is an adaptive response to proteotoxic stresses including heat shock, and is regulated by heat-shock factor 1 (HSF1) in mammals. Proteotoxic stresses challenge all subcellular compartments including the mitochondria. Therefore, there must be close connections between mitochondrial signals and the activity of HSF1. Here, we show that heat shock triggers nuclear translocation of mitochondrial SSBP1, which is involved in replication of mitochondrial DNA, in a manner dependent on the mitochondrial permeability transition pore ANT–VDAC1 complex and direct interaction with HSF1. HSF1 recruits SSBP1 to the promoters of genes encoding cytoplasmic/nuclear and mitochondrial chaperones. HSF1–SSBP1 complex then enhances their induction by facilitating the recruitment of a chromatin-remodelling factor BRG1, and supports cell survival and the maintenance of mitochondrial membrane potential against proteotoxic stresses. These results suggest that the nuclear translocation of mitochondrial SSBP1 is required for the regulation of cytoplasmic/nuclear and mitochondrial proteostasis against proteotoxic stresses.

## Abstract (web summary)

Heat shock induces proteotoxic stress, and the cellular response is mediated by heat-shock factor 1 (HSF1). Here, Tan *et al.* show that following heat shock, mitochondrial SSBP1 translocates to the nucleus and binds HSF1 to enhance the expression of chaperones and support the maintenance of mitochondrial function.

## Graphical abstract

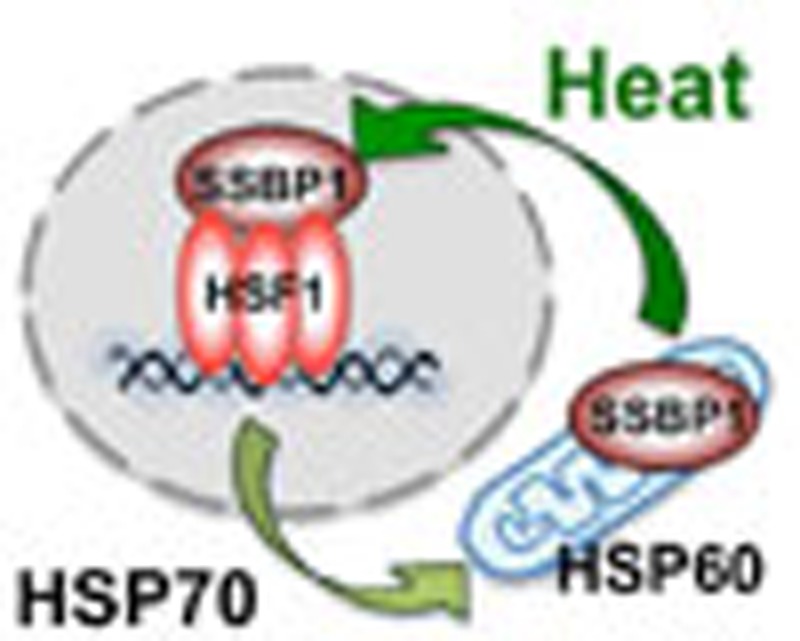

Cells contain a large number of different proteins in distinct subcellular compartments. The proper conformations and physiological concentrations of these proteins, known as protein homeostasis or proteostasis, must be maintained in a variety of environmental and metabolic conditions^1^. To deal with proteotoxic stresses, the cells have evolved sophisticated mechanisms accompanied by changes in gene expression, which adjust proteostasis capacity or buffering capacity against protein misfolding, at the level of protein synthesis, folding and degradation. They include the heat-shock response (HSR) in the cytoplasm/nucleus and the unfolded-protein response (UPR) in the endoplasmic reticulum and mitochondria^2^,^3^,^4^.

The HSR is regulated by heat-shock factor 1 (HSF1) in mammalian cells^5^,^6^. HSF1 mostly stays as an inert monomer in the cytoplasm and nucleus of unstressed cells through the interaction with negative regulators, heat-shock proteins (HSPs) or chaperones^7^,^8^. Heat shock elevates the amount of unfolded and misfolded proteins bound by cytoplasmic/nuclear chaperones including HSP90, HSP70 and HSP40, which is followed by sequestration of these chaperones from HSF1 (ref. 9). As a result, HSF1 accumulates in the nucleus, forms a DNA-binding trimer and binds to the regulatory elements at high levels^10^. HSF1 then recruits coactivators including chromatin remodelling complexes and chromatin-modifying enzymes, and activates target genes including genes encoding for the chaperones^11^.

Heat shock challenges all subcellular compartments including the mitochondria^12^,^13^, which generate energy through oxidative phosphorylation and regulate programmed cell death^14^,^15^. Mitochondria need to communicate with the nucleus to cope with proteotoxic stresses including heat shock^16^,^17^. Mitochondrial chaperones and proteases, which are encoded in the genome, are induced during the accumulation of misfolded proteins within the mitochondrial matrix^18^,^19^. This pathway of mitochondrial UPR is regulated by the transcription factor ATFS-1 in *Caenorhabditis elegans*, which is imported into the mitochondrial matrix under normal conditions and targeted to the nucleus in response to mitochondrial stresses^20^. However, it remains to be determined whether this mechanism is conserved in mammalian cells.

Heat shock induces not only nuclear/cytoplasmic chaperones but also mitochondrial chaperones such as HSP60 and HSP10 (refs 21, 22). Therefore, the pathway of the HSR may be modulated by mitochondrial signals. Here, we demonstrated that heat shock elicited the nuclear translocation of a mitochondrial single-strand DNA-binding protein 1 (SSBP1, also known as mtSSB), which is known to be involved in replication and maintenance of mitochondrial DNA (mtDNA)^23^. SSBP1 was recruited to the promoters of chaperone genes in the nucleus through direct interaction with HSF1, and promoted the expression of chaperones including the mitochondrial chaperones. Furthermore, the HSF1–SSBP1 complex supported cell survival and mitochondrial function in proteotoxic stress conditions.

## Results

### SSBP1 promotes HSP70 expression during heat shock

**Figure 1:**
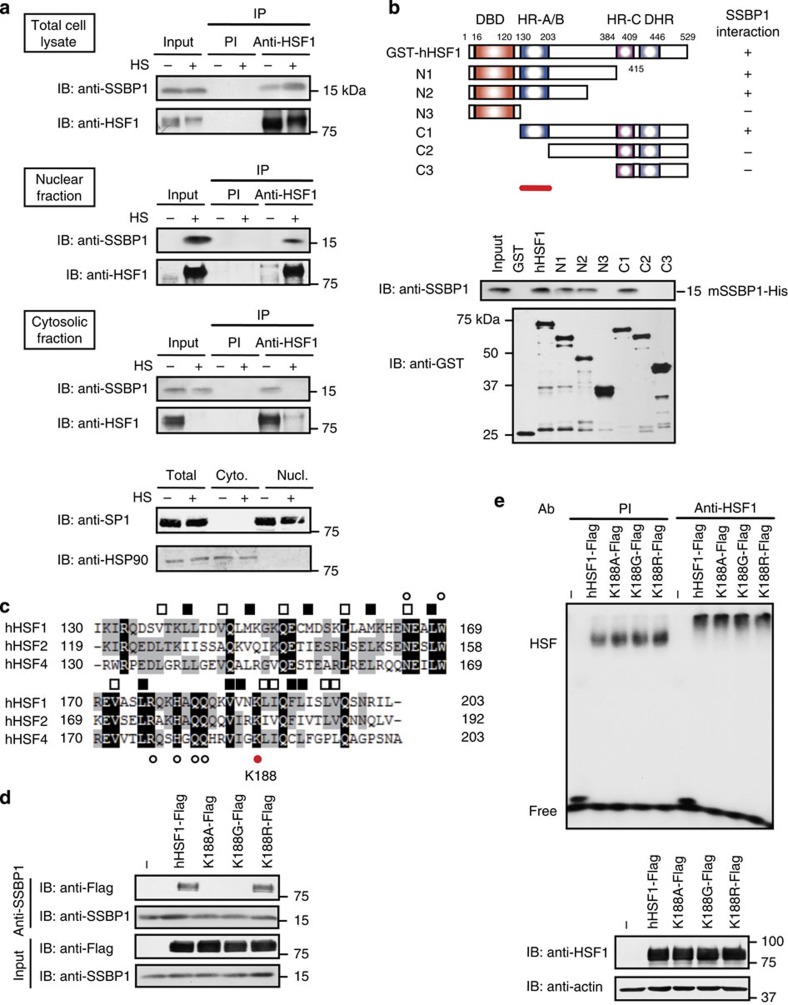
SSBP1 interacts with the trimerization domain of HSF1. (**a**) Interaction between HSF1 and SSBP1. Total cell lysates and the nuclear and cytosolic fractions were prepared from MEF cells at 37 °C (HS-) and cells treated with heat shock at 42 °C for 60 min (HS+). Complexes co-immunoprecipitated using preimmune serum (Pl) or antiserum for HSF1 were blotted with SSBP1 or HSF1 antibody. Total proteins in each lysate were blotted using the same antibodies as a control (Input). They were also blotted using an antibody for a nuclear protein SP1 or HSP90 localized dominantly in the cytoplasm, and are shown at the bottom. (**b**) Trimerization domain of HSF1 is required for the interaction with SSBP1. GST pull-down assay from mixtures of each purified GST-fused hHSF1 protein with mSSBP1-His protein was performed, and blotted with SSBP1 or GST antibody (lower). Degraded recombinant proteins were observed in some lanes. Schematic representation of hHSF1 deletion mutants fused to GST is shown (upper). The ability of each protein to interact with SSBP1 is indicated on the right. DBD, DNA-binding domain; HR, hydrophobic heptad repeat; DHR, downstream of HR-C. A red bar indicates the trimerization domain (HR-A/B, amino acids 130–203), which is required for interaction with SSBP1. (**c**) Alignment of the amino-acid sequences of the trimerization domain of human HSF members. Residues identical among the three sequences are indicated in black, and those identical between two are in grey. Open and solid squares show the heptad repeats of hydrophobic amino acids. Seven residues indicated by dots were mutated (see Supplementary Fig. 1b). Among them, lysine at amino-acid 188 (K188; red dot) was required for the interaction with SSBP1. (**d**) Interaction of SSBP1 with HSF1 point mutants. Each Flag-tagged hHSF1 point mutant at K188 was expressed in HEK293 cells, and complexes co-immunoprecipitated using SSBP1 antibody were blotted with Flag or SSBP1 antibody. (**e**) DNA-binding activity of HSF1 point mutants. Whole-cell extracts were prepared from cells treated as described in **d**, and were subjected to EMSA using ^32^P-labelled HSE-oligonucleotide in the presence of preimmune serum (Pl) or HSF1 antibody (Anti-HSF1) (upper). Western blotting was also performed (lower).

We previously performed a comprehensive identification of proteins interacting with human HSF1, which included mitochondrial SSBP1 (ref. 24). We examined the complex formation of HSF1 and SSBP1 in mouse embryonic fibroblast (MEF) cells, and found that SSBP1 was co-precipitated with HSF1 in total cell lysates, and the co-precipitated SSBP1 level was higher in heat-shocked (at 42 °C for 60 min) cells than that in control cells (Fig. 1a). The co-precipitation of SSBP1 with HSF1 was detected in the nuclear fraction from heat-shocked cells, and also in the cytosolic fraction from control cells, which were free of nuclear contamination. GST pull-down assay showed that recombinant GST fused to human HSF1 (GST-hHSF1) and a polyhistidine-tagged mouse SSBP1 (mSSBP1-His) interacted directly with each other, and the trimerization domain (HR-A/B) of hHSF1 was required for the interaction (Fig. 1b). This domain is evolutionally conserved and can form a triple-stranded, α-helical coiled-coil, through heptad repeats of hydrophobic amino acids^25^. Because SSBP1 also interacted with HSF2 and HSF4 (Supplementary Fig. 1a), we conducted a substitution experiment for seven amino acids in the hHSF1 oligomerization domain that were identical to those in both human HSFs and HSF1 orthologues in various species^6^ (Fig. 1c and Supplementary Fig. 1b). These substitution mutant were overexpressed in HEK293 cells. We found that substitution of lysine at amino-acid 188 (K188) with alanine (A) uniquely abolished the interaction with SSBP1 without affecting its DNA-binding activity (Supplementary Fig. 1c,d). Substitution of K188 with glycine (G), but not with a similar amino-acid arginine (R), also showed the same effect (Fig. 1d,e). These results indicate that SSBP1 interacts with the trimerization domain of hHSF1 through K188 as a contact site.

**Figure 2:**
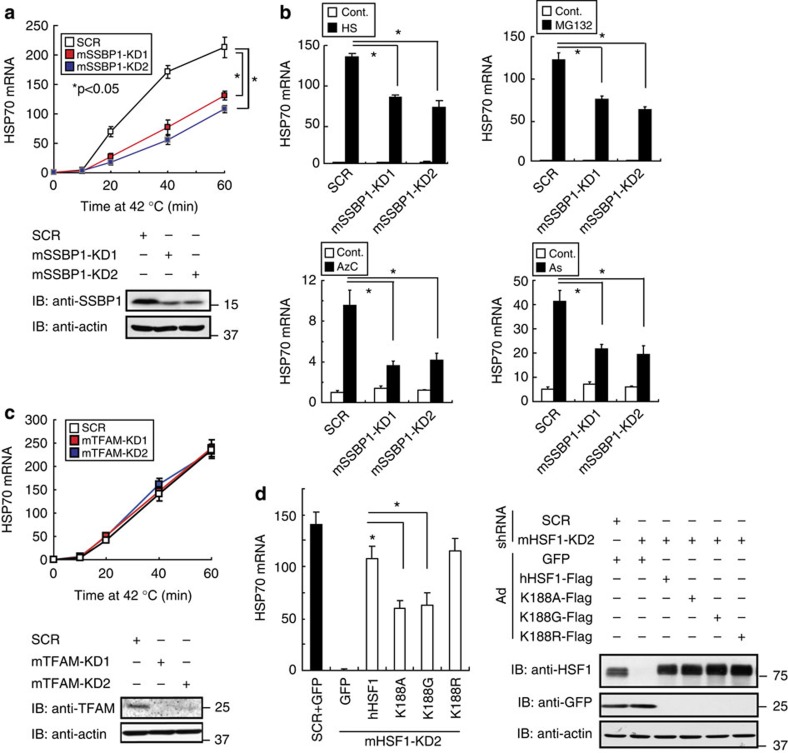
SSBP1 promotes HSP70 expression during heat shock. (**a**) Expression of HSP70 mRNA during heat shock after SSBP1 knockdown. MEF cells were infected with adenovirus expressing scrambled RNA (SCR) or short hairpin RNA (shRNA) against mSSBP1 for 72 h, and heat shocked at 42 °C for the indicated periods. HSP70 mRNA levels were quantified by RT-qPCR, and the levels relative to those in untreated cells are shown (*n*=3). Mean±s.d. is shown. Asterisks indicate *P*<0.05 by analysis of variance. Western blotting was performed to show the levels of SSBP1 and β-actin. (**b**) Expression of HSP70 mRNA in response to various stresses. MEF cells were infected with adenovirus expressing shRNA against mSSBP1 or SCR for 72 h, and were then treated with heat shock at 42 °C for 30 min, MG132 (10 μM) for 3 h, AzC (5 mM) for 6 h or As (50 μM) for 3 h. HSP70 mRNA was quantified by qRT-PCR (*n*=3). Mean±s.d. is shown. Asterisks indicate *P*<0.05 by Student’s *t*-test. (**c**) Expression of HSP70 mRNA after mitochondrial transcription factor A (TFAM) knockdown. MEF cells were infected with adenovirus expressing scrambled RNA (SCR) or shRNA against mTFAM for 72 h, and heat shocked at 42 °C for the indicated periods. HSP70 mRNA was quantified by RT-qPCR (*n*=3). Western blotting was performed to show the levels of TFAM and β-actin. (**d**) Expression of HSP70 mRNA during heat shock in the presence of HSF1 mutants. Endogenous HSF1 was replaced with each HSF1 mutant at K188 or GFP in MEF cells. HSP70 mRNA levels during heat shock at 42 °C for 30 min were quantified by RT-qPCR (*n*=3; left). Mean±s.d. is shown. Asterisks indicate *P*<0.05 by Student’s *t*-test. Cell extracts were also subjected to western blotting (right).

We next examined the impact of SSBP1 on HSP70 expression in MEF cells, and found that knockdown of SSBP1 reduced mRNA levels of HSP70 during heat shock, whereas it did not alter the mRNA level in unstressed conditions (Fig. 2a). SSBP1 knockdown also reduced HSP70 mRNA levels induced by treatment with a proteasome inhibitor MG132, a proline analogue L-azetidine-2-carboxylic acid (AzC), or sodium arsenite (As; Fig. 2b). This effect was not due to impaired replication of mtDNA, because HSP70 expression was not affected by knockdown of the mitochondrial transcription factor A, which is required for the replication and maintenance of mtDNA^26^,^27^ (Fig. 2c). To further exclude the possibility that mitochondrial impairment caused by SSBP1 deficiency indirectly reduced HSP70 expression, we replaced endogenous HSF1 with interaction-deficient mutants. Substitution of endogenous HSF1 with hHSF1-K188A or hHSF1-K188G, but not with hHSF1-K188R, reduced HSP70 expression during heat shock (Fig. 2d). These results demonstrate that mitochondrial SSBP1 promotes the induction of HSP70 expression through direct interaction with HSF1.

### SSBP1 translocates to the nucleus during heat shock

**Figure 3:**
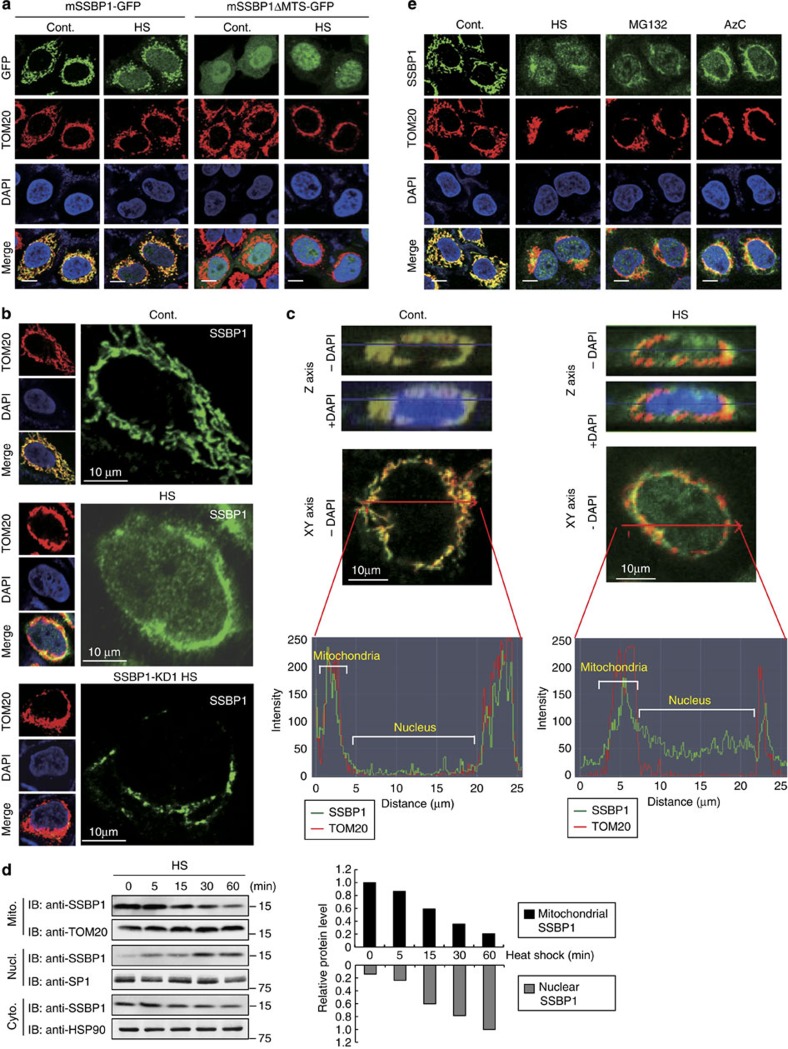
Mitochondrial SSBP1 translocates to the nucleus during heat shock. (**a**) GFP-fused mSSBP1 can accumulate in the nucleus. HeLa cells were transfected with an expression vector for mSSBP1-GFP or mSSBP1ΔMTS-GFP for 48 h, and were treated without (Cont.) or with heat shock at 42 °C for 60 min (HS). They were co-stained with antibody for the mitochondrial marker TOM20, and the nuclear marker DAPI. Each fluorescence image of GFP (green), TOM20 (red) or DAPI (blue) was obtained by scanning confocal microscopy, and merged images (Merge) are shown. Scale bars, 10 μm. (**b**) Nuclear localization of SSBP1 in heat-shocked cells. HeLa cells were treated without (Cont.) or with heat shock at 42 °C for 60 min (HS), and co-stained with antibodies for SSBP1 and TOM20, and DAPI. Each fluorescence image of SSBP1 (green), TOM20 (red) or DAPI (blue) was obtained by scanning confocal microscopy, and merged images (Merge) are shown. Scale bars, 10 μm. Alternatively, HeLa cells were infected with Ad-sh-hSSBP1-KD1 for 72 h, treated with heat shock (SSBP1-KD HS) and co-stained as described above (bottom). (**c**) HeLa cells were treated, co-stained as described in **b**. The fluorescence intensities (arbitrary unit) at the lane (red line) in each cell are shown. (**d**) HeLa cells were treated with heat shock at 42 °C for the indicated periods. Cytoplasmic (Cyto.), nuclear (Nucl.) and mitochondrial (Mito.) fractions were prepared as described in Methods. Equivalent volumes of the fractions were subjected to western blotting using antibody for SSBP1, TOM20, SP1 and HSP90 (left). The signals were estimated using ImageJ, and relative protein levels compared with a control level or that after heat shock were shown (right). (**e**) SSBP1 translocates to the nucleus in response to proteotoxic stresses. HeLa cells were treated without (Cont.) or with heat shock at 42 °C for 60 min (HS), a proteasome inhibitor MG132 (10 μM) for 3 h, or a proline analogue, L-azetidine-2-carboxylic acid (5 mM) for 6 h (AzC). The cells were co-stained as described in **b**. Bars, 10 μm.

HSF1 stays in both the cytoplasm and nucleus in unstressed HeLa cells and predominantly accumulates in the nucleus during heat shock^28^. To interact with HSF1, SSBP1, which localizes in the mitochondria of unstressed cultured cells^29^, may translocate to the cytoplasm or nucleus during heat shock. We overexpressed green fluorescent protein (GFP)-fused full-length or mutated mSSBP1 in HeLa cells, and took photomicrographs of signals of SSBP1 and TOM20, which localized in the outer mitochondrial membrane, using scanning confocal microscopy. mSSBP1-GFP signal was mostly cytoplasmic with some punctate staining and co-localized with TOM20 signal in unstressed cells, indicating that SSBP1 localized in the mitochondria (Fig. 3a). In response to heat shock at 42 °C for 60 min, the signal of TOM20 was concentrated in the perinuclear region, which was consistent with a previous report that mitochondria swelled and migrated towards the perinuclear region during heat shock^30^. Simultaneously, the signal of mSSBP1-GFP appeared in the perinuclear region and slightly in the nucleus. Furthermore, the signal of GFP-fused mSSBP1ΔMTS (148 amino acids), which lacked the mitochondrial targeting sequence (MTS) consisting of 16 amino acids at the N-terminus^31^,^32^, was localized in both the cytoplasm and nucleus of unstressed cells, and it translocated predominantly to the nucleus during heat shock (Fig. 3a). Thus, mitochondrial SSBP1 can translocate to the nucleus during heat shock.

**Figure 4:**
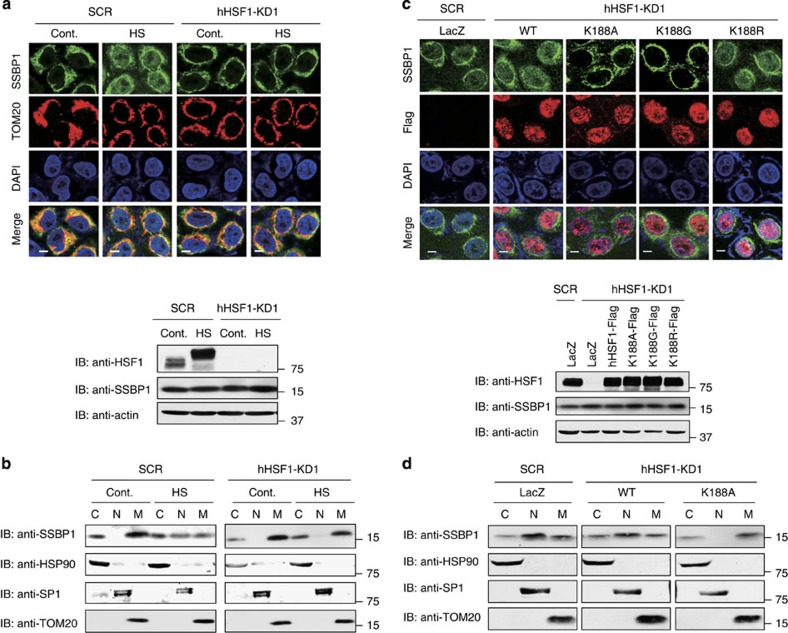
Nuclear translocation of SSBP1 is dependent on HSF1. (**a**) Nuclear translocation of SSBP1 is inhibited by HSF1 knockdown. HeLa cells were infected with Ad-sh-SCR or Ad-sh-hHSF1-KD1 for 72 h, and then treated without (Cont.) or with heat shock at 42 °C for 60 min (HS). The cells were co-stained with antibodies for SSBP1 (green), TOM20 (red) and DAPI (blue), and fluorescence images were merged (Merge; upper). Scale bars, 10 μm. Cell extracts in NP-40 lysis buffer were prepared and subjected to western blotting using an antibody for HSF1 or β-actin (lower). (**b**) Subcellular fractionation. HeLa cells were treated as described in **a**. Cytoplasmic (C), nuclear (N) and mitochondrial (M) fractions were prepared as described in Methods. Equivalent volumes of the fractions were subjected to western blotting using antibodies for SSBP1, HSP90, SP1 and TOM20. (**c**) Subcellular localization of SSBP1 in cells overexpressing HSF1 or its mutant. Cells were infected with adenovirus expressing Ad-sh-hHSF1-KD1 or Ad-sh-SCR for 24 h, and were then infected for 48 h with adenovirus expressing wild-type HSF1 (WT), each point mutant or LacZ. These cells were heat-shocked at 42 °C for 60 min, and co-stained with antibodies for SSBP1 (green) and Flag tag (red), and with DAPI (blue), and fluorescence images were merged (Merge; upper). Cell extracts were prepared and subjected to western blotting using antibody for HSF1 or β-actin (lower). Scale bars, 10 μm. (**d**) Subcellular fractionation was performed using the cells treated as described in **c**, and fractions of the cytoplasm (C), nucleus (N) and mitochondria (M) were subjected to western blotting as described in **b**.

We stained endogenous SSBP1 with SSBP1 antibody, and took photomicrographs of the signal using scanning confocal microscopy. These images confirmed that SSBP1 co-localized in the mitochondrial with TOM20 in control cells, and SSBP1 partially re-localized alone to the nucleus during heat shock at 42 °C for 60 min (Fig. 3b). The signal of SSBP1 was barely detected after heat shock in SSBP1 knockdown cells, indicating that the signal was specific to SSBP1. To create 3D images, we generated 41 separate images of each cell, scanned automatically in the *Z* axis at 0.4 μm intervals. Specific planes from the Z-stacked images are shown (Fig. 3c, upper). Significant nuclear-localized signal from SSBP1 was observed only in heat-shocked cells. We quantified the signals of SSBP1 and TOM20 by measuring the fluorescence intensity on the images in the *X*–*Y* axis (Fig. 3c, lower). SSBP1 signal was detected in the mitochondria, but not in the nucleus, along with TOM20 signal in control cells. In heat-shocked cells, SSBP1 signal, but not that of TOM20, was clearly detected in the nucleus. Furthermore, subcellular fractionation analysis showed that SSBP1 was detected predominantly in the mitochondria and slightly in the cytoplasm of unstressed cells, but not in the nucleus (Fig. 3d). The mitochondrial and cytoplasmic SSBP1 gradually decreased during heat shock at 42 °C until 60 min, whereas a substantial amount of the nuclear SSBP1 appeared upon heat shock. The total protein level of SSBP1 did not change during heat shock (see Fig. 4a). These results indicate that SSBP1 translocates to the nucleus during heat shock, and suggest that the nuclear SSBP1 comes from the mitochondria as well as the cytoplasm.

We examined whether SSBP1 is translocated to the nucleus in response to various stresses. The nuclear translocation of SSBP1 was also observed in response to other proteotoxic stresses such as treatments with a proteasome inhibitor MG132 and the proline analogue AzC (Fig. 3e). In contrast, it was not translocated to the nucleus in response to the stresses, which challenge mitochondrial integrity, such as treatments with paraquat and maneb (inhibitors of mitochondrial respiratory chain complexes I and III, respectively), and FCCP (an uncoupler of oxidative phosphorylation in mitochondria;^33^ Supplementary Fig. 2a). The expression of HSP70 was not induced by these treatments (Supplementary Fig. 2b). Furthermore, the nuclear translocation of SSBP1 and induction of HSP70 expression were not detected in cells treated with hypoxia, which activates hypoxia-inducible factor-1, and hydrogen peroxide, which activates p53 (ref. 34; Supplementary Fig. 2a,b). These results suggest that SSBP1 translocates to the nucleus and affects gene expression specifically on the proteotoxic stress conditions.

### Nuclear translocation of SSBP1 is dependent on HSF1

We examined the time courses of nuclear translocation of SSBP1 and HSF1 in detail. SSBP1 signal in the nucleus faintly appeared 5 min after heat shock and was clearly detected at 15 min (Supplementary Fig. 3a). It then gradually increased during heat shock until 60 min. HSF1 signal, which was obtained using a polyclonal antibody for HSF1, was detected in both the nucleus and cytoplasm in control cells (Supplementary Fig. 3b). HSF1 signal in the cytoplasm also reduced a little 5 min after heat shock and was clearly decreased at 15 min. These results suggest that SSBP1 and HSF1 start to translocate to the nucleus at a similar time point. These cells were further co-stained with the SSBP1 antibody and an HSF1 monoclonal antibody, which recognizes predominantly the nuclear HSF1 in control condition^35^,^36^. The nuclear SSBP1 co-localized with HSF1 in the nucleus of heat-shocked cells, but, unlike HSF1, it did not accumulate at any focus^28^ (Supplementary Fig. 3c).

SSBP1 does not have any putative nuclear localization sequence, so we knocked down HSF1 to reveal its effects on the nuclear translocation of SSBP1. Although the treatment of cells with adenovirus made the TOM20 signal more diffuse in the cytoplasm, SSBP1 still translocated to the nucleus during heat shock (Fig. 4a). Remarkably, the nuclear translocation of SSBP1 was not detected in HSF1 knockdown cells, as shown by the immunofluorescence and subcellular fractionation analyses (Fig. 4a,b). To examine whether the nuclear translocation of SSBP1 requires its interaction with HSF1, we overexpressed wild-type hHSF1 and its mutants in HeLa cells at a level similar to that of endogenous HSF1. SSBP1 was translocated to the nucleus during heat shock in cells expressing wild-type hHSF1 or hHSF1K188R, but not in cells expressing hHSF1K188A or hHSF1K188G (Fig. 4c). Subcellular fractionation analysis of cells expressing wild-type hHSF1 and hHSF1K188A confirmed this result (Fig. 4d). Thus, mitochondrial SSBP1 translocates to the nucleus in a manner that is dependent on its interaction with HSF1.

### ANT–VDAC1 is involved in the nuclear translocation of SSBP1

**Figure 5:**
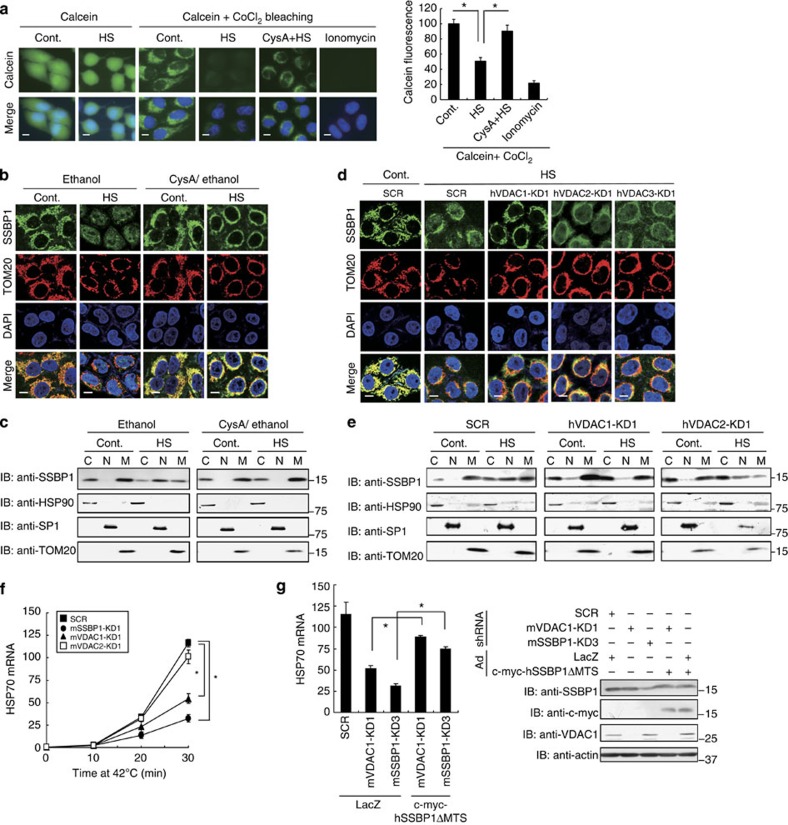
ANT–VDAC1 complex is involved in the nuclear translocation of SSBP1. (**a**) Heat shock-mediated PTP opening is inhibited by cyclosporin A. HeLa cells, pretreated for 2 h with 20 μM cyclosporin A/2% ethanol or 2% ethanol, were treated without (Cont.) or with heat shock at 42 °C for 60 min (HS), and were incubated with calcein AM in the presence or absence of CoCl_2_. Ionomycin was co-incubated as a negative control. Scale bars, 10 μm. The intensity of calcein fluorescence (arbitrary units) from three independent experiments was quantified using ImageJ. Mean±s.d. is shown. Asterisks indicate *P*<0.05 by Student’s *t*-test. (**b**) Cyclosporin A inhibits the nuclear translocation of SSBP1. HeLa cells treated as described in **a** were co-stained with an antibodies for SSBP1 (green) and TOM20 (red), and DAPI (blue), and fluorescence images were merged (Merge). Scale bars, 10 μm. (**c**) Cytoplasmic (C), nuclear (N) and mitochondrial (M) fractions were prepared from cells treated as described in **b**. Equivalent volumes of the fractions were subjected to western blotting using an antibody for SSBP1, HSP90, SP1 and TOM20. (**d**) VDAC1 knockdown inhibits the nuclear translocation of SSBP1. HeLa cells were infected with Ad-sh-SCR or adenovirus expressing short hairpin RNA (shRNA) for each VDAC for 72 h, and then treated without (Cont.) or with heat shock at 42 °C for 60 min (HS). These cells were co-stained as described in **b**. Scale bars, 10 μm. (**e**) Cells treated as described in **d** were subjected to subcellular fractionation as described in **c**. (**f**) Expression of HSP70 in VDAC knockdown cells. MEF cells were infected with Ad-sh-SCR or adenovirus expressing shRNA for mouse VDAC1, VDAC2 or SSBP1 for 72 h, and heat shocked at 42 °C for the indicated periods. HSP70 mRNA was quantified by RT-qPCR (*n*=3). Mean±s.d. is shown. Asterisks indicate *P*<0.05 by analysis of variance. (**g**) Restoration of HSP70 expression by the overexpression of human SSBP1ΔMTS in VDAC1 knockdown cells. VDAC1 or SSBP1 was knocked down in cells, and then c-myc-tagged hSSBP1ΔMTS was overexpressed. HSP70 mRNA levels during heat shock at 42 °C for 30 min were quantified by RT-qPCR (*n*=3; left). Mean±s.d. is shown. Asterisks indicate *P*<0.05 by Student’s *t*-test. Cell extracts were subjected to western blotting (right).

Mitochondrial membrane potential is an important indicator of functional mitochondria, and is reduced in response to proteotoxic stresses such as heat shock and proteasome inhibition^12^,^37^. The reduction of this membrane potential may be the result of increased mitochondrial permeability transition^15^. To understand the regulation of nuclear translocation of mitochondrial SSBP1, we examined roles of the mitochondrial permeability transition pore (PTP), which is one of complexes connecting the mitochondrial matrix to the cytoplasm^38^. Mitochondrial PTP consists of the voltage-dependent anion channel (VDAC) and adenine nucleotide translocase (ANT), which is associated with cyclophilin D^15^. First, we treated cells with cyclosporin A, which blocks mitochondrial PTP opening by inhibiting the activity of cyclophilin D^15^. Heat shock induced mitochondrial PTP opening, and this opening was inhibited by the pretreatment with cyclosporin A (Fig. 5a). Cyclosporin A further inhibited the nuclear translocation of SSBP1 during heat shock (Fig. 5b,c). VDACs are not essential for the PTP opening^38^,^39^, but are required for dissipation of mitochondrial membrane potential in some conditions^40^,^41^,^42^. Therefore, we next knocked down VDACs, and found that VDAC1 knockdown markedly suppressed mitochondrial PTP opening in response to heat shock, whereas the knockdown of VDAC2 or VDAC3 suppressed it only slightly (Supplementary Fig. 4a,b). In accordance with this, the knockdown of VDAC1, but not that of VDAC2 or VDAC3, inhibited the heat shock-induced nuclear translocation of SSBP1 (Fig. 5d,e). Thus, the ANT–VDAC1 complex is involved in the nuclear translocation of SSBP1 during heat shock.

The PTP opening may trigger cell death in addition to the promotion of the nuclear translocation of SSBP1 (refs 15, 34). When HeLa cells were exposed to extreme heat shock at 45 °C for 2 h, they were induced to die (Supplementary Fig. 5a). Simultaneously, apoptotic factors such as cytochrome *c* (Cyto *c*) and apoptosis-inducing factor (AIF) that localize in the mitochondrial intermembrane space under normal conditions were released, and caspase-3 was cleaved and activated (Supplementary Fig. 5b). However, the cells did not die when they were exposed to moderate heat shock at 42 °C for 1 h as described above, and the release of Cyto *c* and AIF and cleavage of caspase-3 were not detected (Supplementary Fig. 5a,b).

We next examined whether the nuclear translocation of SSBP1 is associated with the induction of HSP70 mRNA in MEF cells. It was revealed that the knockdown of VDAC1, but not that of VDAC2, markedly reduced the expression of HSP70 mRNA during heat shock (Fig. 5f and Supplementary Fig. 4a). Furthermore, the overexpression of hSSBP1ΔMTS restored HSP70 expression in VDAC1 knockdown cells as well as in SSBP1 knockdown cells (Fig. 5g). These results indicate that the nuclear translocation of SSBP1 is associated with the expression of HSP70 during heat shock.

### SSBP1 promotes the recruitment of BRG1

**Figure 6:**
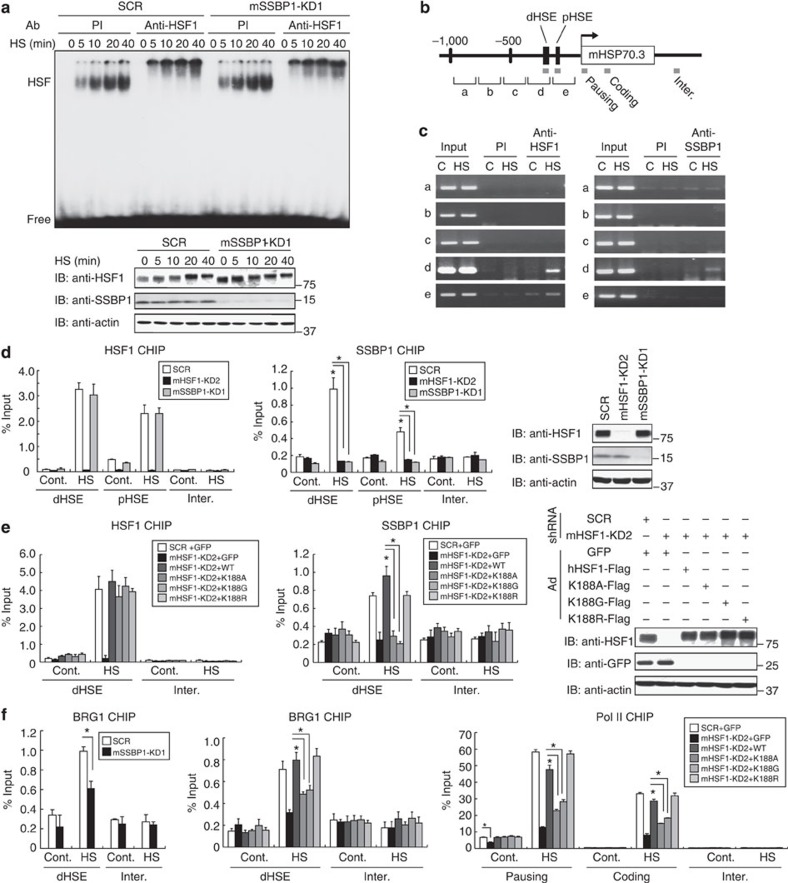
HSF1–SSBP1 complex promotes recruitment of BRG1. (**a**) SSBP1 does not affect DNA-binding activity of HSF1. MEF cells were infected for 72 h with Ad-sh-mSSBP1-KD1 or Ad-sh-SCR, and heat-shocked at 42 °C until 40 min (HS). Whole-cell extracts were prepared and subjected to EMSA using a ^32^P-labelled ideal HSE-oligonucleotide in the presence of preimmune serum (PI) or HSF1 antibody (Anti-HSF1; upper). Western blotting was performed (lower). (**b**) Schematic representation of mouse HSP70.3 (HSPA1A) locus. Regions **a**–**e** in the HSP70.3 promoter were amplified by PCR using primer sets. Amplified proximal and distal HSE regions (pHSE and dHSE), and coding (coding) and intergenic regions (inter.) by real-time PCR are shown as grey boxes. (**c**) SSBP1 is recruited to dHSE containing region. ChIP-enriched DNA was prepared, using preimmune serum (PI) or antibody for HSF1 or SSBP1, from MEF cells untreated (C) or treated with heat shock at 42 °C for 30 min (HS). DNA fragments of the regions were amplified by PCR. (**d**) HSF1-dependent recruitment of SSBP1. MEF cells, in which HSF1 or SSBP1 was knocked down, were treated without (Cont.) or with heat shock at 42 °C for 30 min (HS). ChIP-qPCR analyses were performed in pHSE, dHSE and intergenic (Inter.) regions using HSF1 or SSBP1 antibody (*n*=3). Mean±s.d. is shown. Asterisks indicate *P*<0.05 by Student’s *t*-test. HSF1 and SSBP1 protein levels were examined by western blotting. (**e**) SSBP1 occupancy in the presence of HSF1 mutants. Cells, in which endogenous HSF1 was substituted with each interaction mutant, were untreated (Cont.) or treated with heat shock at 42 °C for 30 min (HS). ChIP-qPCR analyses were performed (*n*=3). Protein levels of HSF1 and its mutants were examined by western blotting. (**f**) SSBP1 promotes the recruitment of BRG1 and Pol II. SSBP1 knockdown cells were heat-shocked as described in **d**, and ChIP-qPCR analyses were performed using BRG1 antibody (*n*=3). Endogenous HSF1 was also substituted with each interaction mutant as described in **e**. ChIP-qPCR analyses in the dHSE and pausing, coding and intergenic regions were performed using BRG1 or Pol II antibody (*n*=3).

To reveal the mechanisms by which nuclear SSBP1 promoted HSP70 expression, we analysed the effects of SSBP1 on HSF1 binding to DNA *in vitro* and *in vivo*. Electrophoretic mobility shift assay (EMSA) showed that the DNA-binding activity of HSF1 was induced during heat shock at 42 °C at similar levels in scrambled RNA-treated and SSBP1 knockdown cells (Fig. 6a). Surprisingly, chromatin immunoprecipitation (ChIP) assay showed that not only HSF1 but also SSBP1 occupied mouse HSP70.3 (HSPA1A) promoter during heat shock *in vivo* (Fig. 6b,c). The occupancy of SSBP1 was detected only on the region containing distal heat shock element (dHSE) within the promoter (−937 to +75). We performed ChIP-quantitative PCR (qPCR) analysis using primer sets for dHSE or proximal HSE (pHSE), and found that binding of HSF1 to the HSP70.3 promoter increased during heat shock at similar levels in both scrambled RNA-treated and SSBP1 knockdown cells (Fig. 6d). SSBP1 knockdown does not affect the recruitment of HSF1 to the promoter. SSBP1 occupied the HSP70.3 promoter (with a strong signal at dHSE) during heat shock, and the knockdown of HSF1 abolished the occupancy of SSBP1. Furthermore, SSBP1 did not occupy the HSP70.3 promoter when endogenous HSF1 was replaced with its interaction mutants hHSF1-K188A or hHSF1-K188G, but not with hHSF1-K188R (Fig. 6e). The occupancy of SSBP1 on the HSP70.3 promoter did not require its ability to bind to single-stranded DNA (ssDNA), because SSBP1ΔMTS-W68T/F74A, which could not bind to ssDNA^43^, was recruited to the promoter (Supplementary Fig. 6). These results demonstrate that HSF1 directly recruits SSBP1 to the HSP70.3 promoter during heat shock.

The HSF1-transcription complex includes coactivators such as BRG1-containing chromatin remodelling complex, which promotes transcription of the *HSP70* gene^10^,^11^. The recruitment of BRG1 is tightly correlated with the expression of HSPs^44^,^45^. Therefore, we examined the recruitment of BRG1 to the HSP70.3 promoter. It was revealed that the knockdown of SSBP1 or replacement with each interaction mutant of HSF1 reduced the recruitment of BRG1 during heat shock (Fig. 6f). Consistently, the recruitment of RNA polymerase II (Pol II) was also reduced in the presence of these interaction mutants. Thus, SSBP1 promotes the recruitment of BRG1 to the HSP70.3 promoter during heat shock.

### SSBP1 enhances chaperone expression during heat shock

**Figure 7:**
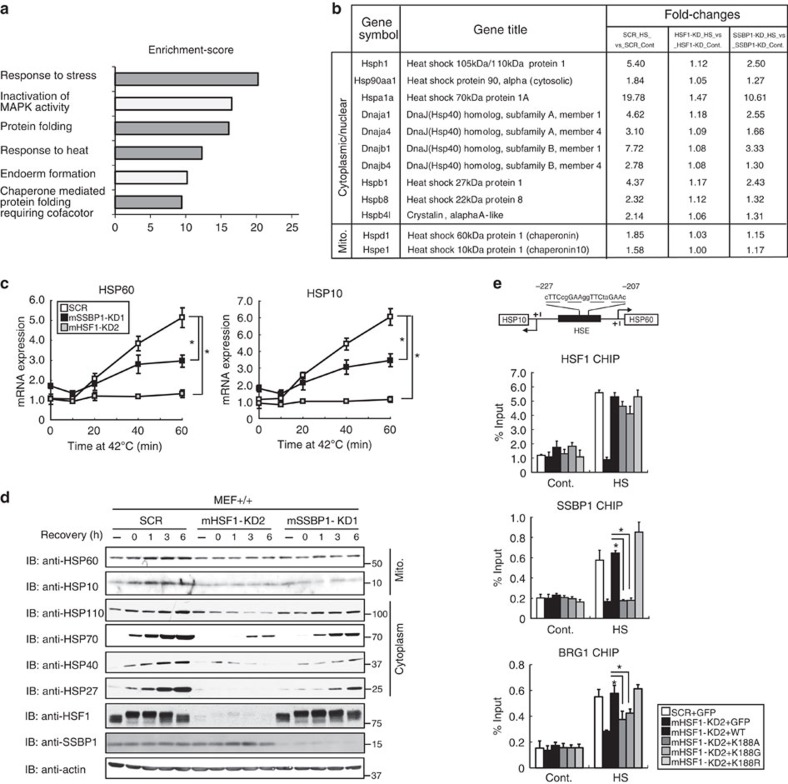
SSBP1 enhances the expression of mitochondrial and cytoplasmic/nuclear chaperones. (**a**) Chaperone-related terms are over-represented in gene ontology enrichment analysis of heat-inducible genes. MEF cells, which were infected for 72 h with Ad-sh-mHSF1-KD2, Ad-sh-mSSBP1-KD1 or Ad-sh-SCR, were untreated (C) or treated with heat shock at 42 °C for 1 h (HS). Microarray analysis was performed using total RNA isolated from these cells. We identified 154 heat-inducible genes (fold change>+1.5, *P*<0.05; *n*=3) in scrambled RNA-treated cells, and this gene set was subjected to gene ontology enrichment (Partek Genomics Suite 6.5). The enriched biological process categories (*P*<0.0001, determined by Fisher’s exact test) are shown. (**b**) Summary of heat-inducible chaperone genes. All cytoplasmic/nuclear chaperones and mitochondrial chaperones among 154 heat-inducible genes from **a** are shown. Fold-changes (HS versus Cont.) were calculated by comparing mRNA levels in heat-shocked cells with those at control levels in HSF1 or SSBP1 knockdown cells (*n*=3). (**c**) Expression of mitochondrial chaperones during heat shock in SSBP1 or HSF1 knockdown cells. MEF cells were infected with Ad-sh-SCR, Ad-sh-mSSBP1-KD1 or Ad-sh-mHSF1-KD2, and heat shocked at 42 °C for the indicated periods. HSP60 and HSP10 mRNAs were quantified by RT-qPCR (*n*=3). Mean±s.d. is shown. Asterisks indicate *P*<0.05 by analysis of variance. (**d**) Protein levels of mitochondrial as well as cytoplasmic/nuclear chaperones in SSBP1 knockdown cells. Cells infected with Ad-sh-SCR, Ad-sh-mSSBP1-KD1 or Ad-sh-mHSF1-KD2, were heat shocked at 42 °C for 1 h and then allowed to recover for the indicated periods. Western blotting was performed. (**e**) HSF1–SSBP1 complex promotes the recruitment of BRG1 to the HSP60/HSP10 promoter. Cells, in which endogenous HSF1 was replaced with each interaction mutant, were untreated (Cont.) or treated with heat shock at 42 °C for 30 min (HS). ChIP-qPCR analyses in HSP60/HSP10 promoter were performed (*n*=3). Mean±s.d. is shown. Asterisks indicate *P*<0.05 by Student’s *t*-test. In schemes of promoter regions, each HSE consensus sequence, nGAAn, is underlined.

To identify genes regulated by the HSF1–SSBP1 complex, we performed microarray analysis using MEF cells treated with or without heat shock at 42 °C for 1 h, and identified 154 heat-inducible genes (fold change >1.5). Gene ontology enrichment analysis of this gene set revealed over-represented terms related to chaperones (Fig. 7a, grey bars). Among the products of all chaperone genes, ten were cytoplasmic/nuclear chaperones, such as HSP110, HSP90, HSP70, HSP40s and small HSPs, and two were the mitochondrial chaperones, HSP60 and HSP10 (Fig. 7b). The fold inductions of these genes seemed to decrease with SSBP1 knockdown. We confirmed that the mRNA levels of mitochondrial HSP60 and HSP10 were induced modestly (approximately five- to sixfold) during heat shock (Fig. 7c), whereas those of cytoplasmic/nuclear chaperones were robustly induced (more than 25-fold) (Supplementary Fig. 7a). The fold inductions of all these chaperones decreased by half after heat shock at 42 °C for 60 min with SSBP1 knockdown, whereas they were hardly induced with HSF1 knockdown (Fig. 7c and Supplementary Fig. 7a). The protein levels of HSP60, HSP10, HSP110, HSP70 and HSP25 were correlated with each mRNA level (Fig. 7d). We performed ChIP assay and found that SSBP1 was recruited to the common HSE in the *HSP60* and *HSP10* genes as well as to the promoters of cytoplasmic/nuclear chaperones, but was not recruited in the presence of the interaction mutants hHSF1K188A and hHSF1-K188G (Fig. 7e and Supplementary Fig. 7b). HSF1 mutants that cannot bind to SSBP1 were able to occupy these promoters, suggesting that SSBP1 does not direct HSF1 to its targets. Furthermore, the recruitment of BRG1 was reduced in the presence of these interaction mutants. Among mitochondrial chaperone genes, *mtHSP70* gene has been shown to be bound by HSF1 in heat-shocked human and mouse cells^45^,^46^,^47^. We found that the induction of mtHSP70 expression during heat shock was strictly dependent on SSBP1 as well as HSF1, and the recruitment of BRG1 was also reduced in the presence of the HSF1 mutants (Supplementary Fig. 7c,d). Thus, SSBP1 enhances the expression of mitochondrial and cytoplasmic/nuclear chaperones, in part by facilitating the formation of the HSF1-transcription complex including BRG1.

### HSF1–SSBP1 complex protects cells from proteotoxic stresses

**Figure 8:**
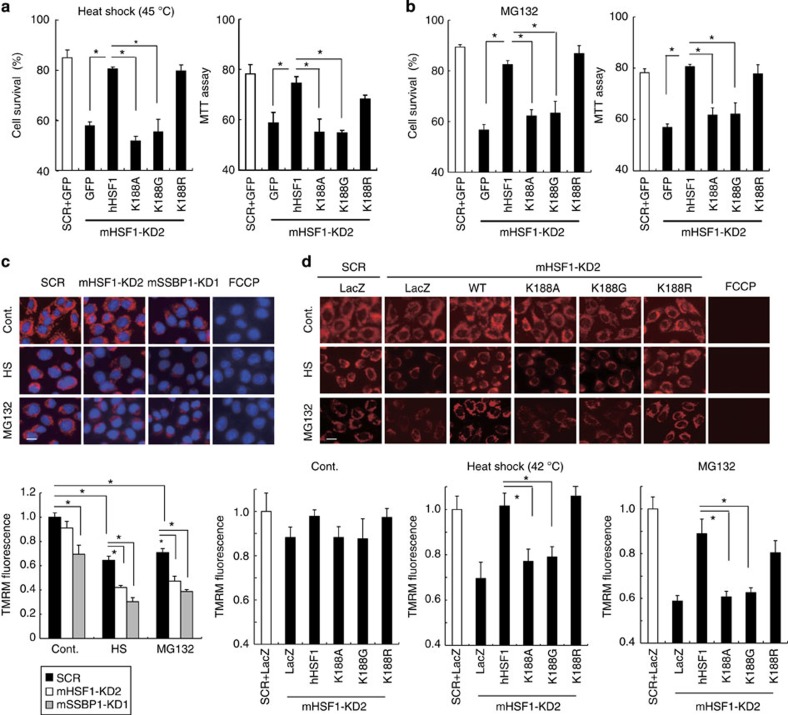
HSF1–SSBP1 complex supports cell survival and mitochondrial function. (**a**) HSF1–SSBP1 complex protects cells from heat shock. MEF cells (HSF1+/+, clone #10) were infected with Ad-sh-mHSF1-KD2 or Ad-sh-SCR for 24 h, and then infected with adenovirus expressing wild-type hHSF1, each hHSF1 mutant or GFP for 48 h. After incubating the cells at 45 °C for 2 h, the number of viable cells excluding trypan blue was counted (left) and thiazolyl blue tetrazolium bromide (MTT) assay was performed (right; *n*=3). Mean±s.d. is shown. Asterisks indicate *P*<0.05 by Student’s *t*-test. (**b**) HSF1–SSBP1 complex protects cells from a proteasome inhibitor. MEF cells were treated as described in **a**, and were incubated with MG132 (10 μM) for 6 h. The number of viable cells was counted (left) and MTT assay was performed (right; *n*=3). (**c**) Heat shock and proteasome inhibition reduce mitochondrial membrane potential. MEF cells were infected with Ad-sh-SCR, Ad-sh-mHSF1-KD2 or Ad-sh-mSSBP1-KD1 for 72 h. After being incubated at 42 °C for 1 h or with MG132 for 6 h, the cells were stained for 30 min at 37 °C with DAPI (blue) and a fluorescent dye TMRM (red) that accumulates in the mitochondria in a manner dependent on membrane potential. FCCP (10 μM) was added for 10 min as a negative control. Scale bars, 20 μm. Representative fluorescence images are shown, and TMRM fluorescence intensities (arbitrary units) are compared with that in unstressed scrambled RNA-treated cells were quantified using ImageJ (*n*=3). Mean±s.d. is shown. Asterisks indicate *P*<0.05 by Student’s *t*-test. (**d**) HSF1–SSBP1 complex supports maintenance of mitochondrial membrane potential in proteotoxic conditions. Cells were infected with Ad-sh-mHSF1-KD2 or Ad-sh-SCR for 24 h, and then infected with adenovirus expressing wild-type hHSF1, each hHSF1 mutant or LacZ for 48 h. After being incubated at 42 °C for 1 h or with MG132 for 6 h, the cells were stained with TMRM in the absence or presence of FCCP as described in **c**. Scale bars, 20 μm. TMRM fluorescence intensities (arbitrary units) compared with that in scrambled RNA-treated cells (white bars) were quantified using ImageJ (*n*=3).

The reduced expression of a set of chaperones may affect cell survival in proteotoxic stress conditions. We examined the survival of MEF cells during extreme heat shock at 45 °C. We showed that cell survival during heat shock was markedly reduced when HSF1 was knocked down, and was restored by the overexpression of wild-type hHSF1 or hHSF1-K188R (Fig. 8a). However, it was not restored by overexpression of the interaction mutants, hHSF1-K188A or hHSF1-K188G. Furthermore, the survival of cells during treatment with MG132 was also reduced in HSF1-knockdown cells, and was restored by the overexpression of hHSF1, but not that of the interaction mutants (Fig. 8b). Thus, the interaction between HSF1 and SSBP1 affected the survival of cells on proteotoxic stress conditions more strongly than we expected from the fact that the transcriptional activity of the interaction mutant of HSF1 was reduced partially.

Mitochondrial chaperones such as HSP60 and HSP10 control apoptosis, mitochondrial membrane potential and PTP opening^48^,^49^,^50^. Therefore, we examined mitochondrial membrane potential, which regulates cell death and energy production, using a fluorescent probe tetramethylrhodamine methyl ester (TMRM). The intensity of TMRM fluorescence was not affected by scramble RNA-treated cells or HSF1 knockdown cells in unstressed conditions, but was moderately reduced in SSBP1 knockdown cells (Fig. 8c). The fluorescence intensity was moderately reduced during heat shock at 42 °C for 1 h or with MG132 for 6 h in scrambled RNA-treated cells^12^,^37^. It was much more reduced under these stressed conditions in HSF1 or SSBP1 knockdown cells than in scrambled RNA-treated cells. We replaced endogenous HSF1 with its mutants and examined the membrane potential. The intensity of TMRM fluorescence in HSF1-knockdown cells was reduced by heat shock or MG132 treatment, but the reduced fluorescence intensities were restored by overexpression of wild-type hHSF1 or hHSF1-K188R, but were not by that of hHSF1-K188A or hHSF1-K188G (Fig. 8d). These results indicate that HSF1–SSBP1 complex supports the maintenance of mitochondrial membrane potential in proteotoxic stress conditions.

## Discussion

The HSR was originally discovered as the induction of a set of puffs in salivary gland chromosomes of *Drosophila* after treatment with heat shock or dinitrophenol, an uncoupler of oxidative phosphorylation^51^. Thereafter, many agents that interfere with mitochondrial function, including uncouplers of oxidative phosphorylation and inhibitors of electron transport, were shown to induce the HSR in *Drosophila*^52^. These observations suggested that mitochondria were the primary target of many different stimuli that induce the HSR, and this response protected cells from respiratory stress. However, these hypotheses were not well supported by the fact that the HSPs induced by heat shock were not concentrated in the mitochondria, and respiration-deficient yeast mutants still produced HSPs in response to heat shock^53^. We now understand that the HSR is triggered by the elevation of misfolded proteins within cells and the dissociation of chaperones from HSF1 (ref. 9). Nevertheless, heat shock induces mitochondrial chaperones such as HSP60 and HSP10 (refs 21, 22), and the HSR is modulated by mitochondrial signals such as increased levels of reactive oxygen species, which are produced in excess from impaired mitochondria^54^. In fact, the activity of HSF1 is regulated by cellular redox status through its cysteine residues^55^,^56^. However, the mitochondrial factor that directly links signals in the mitochondria to the HSR remained unknown. Here, we demonstrate that mitochondrial SSBP1 promotes the induction of cytoplasmic/nuclear and mitochondrial chaperones (Figs 2 and 7), and supports cell survival and the maintenance of mitochondrial membrane potential, an indicator of functional mitochondria, in proteotoxic stress conditions (Fig. 8).

SSBP1 is a homologue of *Escherichia coli* SSB and plays roles in mtDNA metabolism including replication by binding to ssDNA^23^. Thus, its function is analogous to that of replication protein A (RPA), which is also an interacting partner of HSF1, in the nucleus^24^. The localization of SSBP1 has been thought to be mitochondrial under physiological conditions, and viral infection was suggested to induce the nuclear translocation of SSBP1. In Epstein–Bar virus-infected cells, overexpression of the viral-encoded protein Zta, which is a basic leucine zipper DNA-binding protein, induced SSBP1 to enter the nucleus^57^. We show that SSBP1 translocates to the nucleus when cells are exposed to proteotoxic stresses including treatment with heat shock, proteasome inhibitor or proline analogue (Fig. 3). The nuclear translocation of SSBP1 is dependent on its interaction with the oligomerization domain of HSF1 (Figs 1 and 4), suggesting that HSF1 transports SSBP1 to the nucleus. Furthermore, the HSF1–SSBP1 complex on the promoters potentiates HSF1 transcriptional activity by facilitating the recruitment of BRG1, a component of chromatin remodelling complexes (Figs 6 and 7). Thus, we elucidate a new role of SSBP1 in the regulation of gene expression in the nucleus.

The PTP controls mitochondrial homeostasis via a sudden increase in membrane permeability in response to stimuli, and is important for mitochondrial functions under various pathophysiological conditions^58^. Opening of the PTP is associated with the loss of inner membrane potential required for energy production and possibly the release of cell death-related factors from the intermembrane space such as Cyto *c*, AIF and endonuclease G^15^,^34^. Previous studies suggested that proteotoxic stresses including heat shock and proteasome inhibition might open the PTP^12^,^37^. We show that pretreatment with cyclosporine A inhibits its opening and the nuclear translocation of SSBP1 during heat shock (Fig. 5a–c). VDACs are not always essential for opening of the PTP^38^,^39^,^40^,^41^,^42^. However, VDAC1 is required for heat shock-mediated PTP opening and the nuclear translocation of SSBP1 (Fig. 5d,e and Supplementary Fig. 4b). Thus, the mitochondrial PTP ANT–VDAC1 complex is required for the nuclear translocation of SSBP1. Remarkably, the PTP opening induces the pro-survival function of SSBP1 (refs 12, 59) without the release of pro-apoptotic factors such as Cyto *c* and AIF in response to moderate heat shock (Supplementary Fig. 5). Recently, it was reported that the pyruvate dehydrogenase complex in mitochondria translocates to the nucleus and provides acetyl-CoA required for histone acetylation and epigenetic regulation^60^. Although a mechanism that can explain the specific export of these proteins across the mitochondrial membrane remains unknown, the mitochondrial expatriate may be involved more generally in the regulation of genomes in response to stimuli.

Proteostasis in mitochondria is maintained by the mitochondrial UPR, which is characterized by the induction of mitochondrial chaperones, such as HSP60, HSP10 and mtHSP70, and proteases, such as Lon and ClpP^3^. Although this response is regulated by ATFS-1 in *C. elegans*^16^,^17^, a mammalian homologue of ATFS-1 has not yet been identified. We clearly show that HSF1 induces the expression of HSP60, HSP10 and mtHSP70 during heat shock in mouse cells, and SSBP1, which localizes mostly in the mitochondria, enhances their expression by potentiating the transcriptional activity of HSF1 (Figs 6, 7). Thus, the HSF1–SSBP1 complex is involved in the mitochondrial UPR during proteotoxic stresses at least in mammalian cells. Unexpectedly, the effects of HSF1 on cell survival against proteotoxic stresses are largely dependent on SSBP1, partly through the maintenance of mitochondrial function (Fig. 8). Even in physiological conditions, HSF1 is required for mitochondrial homeostasis in some tissues, including the heart^61^, and for the expression of HSP60 in the olfactory epithelium^62^. Because the induction of mitochondrial UPR is associated with longevity in *C. elegans* and *Drosophila*^63^,^64^, the strong effects of HSF on ageing and the age-related misfolding diseases^65^,^66^,^67^,^68^ may be in part due to its role in mitochondrial proteostasis.

## Methods

### Cell cultures and treatments

MEFs^67^, HEK293 and HeLa cells (American Type Culture Collection) were maintained at 37 °C in 5% CO_2_ in Dulbecco’s modified Eagle’s medium containing 10% fetal bovine serum. Cells were treated with heat shock at 42 °C or reagents such as MG132 (10 μM), AzC (5 mM) and sodium arsenite (As, 50 μM).

### Assessment of mRNA

Immortalized MEF cells (stock #10) were infected with an adenovirus expressing each short hairpin RNA (1 × 10^8^ p.f.u. per ml) for 2 h, maintained with normal medium for 70 h and treated with heat shock or reagents as described above. To replace endogenous HSF1 with HSF1 mutants in MEF cells, cells were infected with Ad-sh-mHSF1-KD2 (1 × 10^8^ p.f.u. per ml) for 2 h and maintained in normal medium for 22 h. They were then infected with an adenovirus expressing an hHSF1 mutant (1 to 5 × 10^7^ p.f.u. per ml) for 2 h and maintained with normal medium for a further 46 h. Total RNA was extracted and mRNA levels were estimated by reverse transcription (RT)–qPCR using primers listed in Supplementary Table 1. Relative quantities of mRNAs were normalized against S18 ribosomal RNA levels. To knockdown VDAC1 or SSBP1 and overexpress c-myc-tagged hSSBP1ΔMTS, cells were infected with Ad-sh-mVDAC1-KD1 or Ad-sh-mSSBP1-KD3 that targets the 5′ untranslated region of mSSBP1 mRNA (1 × 10^8^ p.f.u. per ml) for 2 h and maintained in normal medium for 22 h. They were then infected with pAd-c-myc-hSSBP1ΔMTS (5 × 10^7^ p.f.u. per ml) for 2 h and maintained with normal medium for a further 46 h. Total RNA was extracted and mRNA levels were estimated as described above.

### Co-immunoprecipitation

We generated a rabbit antiserum against mouse SSBP1 (αmSSBP1-1) by immunizing rabbits with bacterially expressed recombinant mSSBP1-His. MEF cells treated with or without heat shock at 42 °C for 60 min were lysed with NP-40 lysis buffer containing 1.0% Nonidet P-40, 150 mM NaCl, 50 mM Tris-HCl (pH 8.0), 1 μg ml^−1^ leupeptin, 1 μg ml^−1^ pepstatin A and 1 mM phenylmethylsulfonyl fluoride. After centrifugation, the supernatant containing 4 mg proteins was incubated with 3 μl of rabbit polyclonal antibody for HSF1 (αmHSF1j, ABE1044, Millipore) or preimmune serum at 4 °C for 16 h, and mixed with 40 μl protein A-Sepharose beads (GE Healthcare) by rotating at 4 °C for 1 h. The complexes were washed five times with NP-40 lysis buffer, and were subjected to western blotting using rabbit polyclonal antibody for SSBP1 (αmSSBP1-1) or HSF1 (αmHSF1j). Alternatively, the immunoprecipitation was performed using the cytosolic fractions (see below) containing 4 mg proteins. The uncropped scanned full gels are shown in Supplementary Fig. 8.

cDNAs for Flag-tagged hHSF1 and hHSF1 point mutants were created by RT–PCR, and were inserted into pShuttle-CMV vector (Agilent Technologies) at *Kpn*I/*Xho*I sites (pShuttle-hHSF1-Flag, pShuttle-hHSF1-K188A and so on). Sequences were verified using the 3500 Genetic Analyzer (Applied Biosystems). Viral DNAs and viruses (Ad-hHSF1-Flag, Ad-hHSF1-K188A, etc) were generated according to the manufacturer’s instructions for the AdEasy adenoviral vector system (Agilent Technologies)^24^. HEK293 cells in a 10-cm dish were transfected with a pShuttle-CMV expression vector for Flag-tagged hHSF1 or an hHSF1 point mutant, and were lysed with NP-40 lysis buffer. After centrifugation, the supernatant (400 μl) was incubated with 3 μl of rabbit antiserum for SSBP1 (αmSSBP1-1) at 4 °C for 1 h, and mixed with 40 μl protein A- or protein G-Sepharose beads (GE Healthcare) by rotating at 4 °C for 1 h. The complexes were washed with NP-40 lysis buffer, and were subjected to western blotting using mouse monoclonal IgG for Flag peptide (M2, Sigma-Aldrich).

### Immunofluorescence

HeLa cells cultured on glass coverslips in 6 cm dishes at 37 °C for 24 h were infected for 2 h with adenovirus expressing scrambled RNA or short hairpin RNA for HSF1 or each VDAC protein (1 × 10^7^ p.f.u. per ml), and then maintained with normal medium for 70 h. After being heat-shocked at 42 °C for 60 min, the cells were fixed with 4% paraformaldehyde in medium at room temperature for 10 min. They were washed with PBS, permeabilized for 10 min with 0.2% Triton X-100/PBS, blocked in 2% non-fat dry milk/PBS for 1 h at room temperature, incubated with 1:200 diluted rabbit serum for SSBP1 (αmSSBP1-1) and 1:100 diluted mouse IgG for TOM20 (sc-17764, Santa Cruz) or rat IgG for HSF1 (ab61382, Abcam) in 2% milk/PBS at 4 °C overnight, and then incubated with FITC-conjugated goat anti-rabbit IgG (1:200 dilution; Cappel) or Alexa Fluor 568-conjugated goat anti-mouse IgG or Alexa Fluor 546-conjugated goat anti-rat IgG (1:200 dilution; Molecular Probes). The coverslips were washed and mounted in a VECTASHIELD with 4′-6-diamidino-2-phenylindole (DAPI) mounting medium (Vector Laboratories). Fluorescence images were taken using Axiovert 200 fluorescence microscope (Carl Zeiss) or LSM510 META confocal microscope (Carl Zeiss). To analyse SSBP1 localization in the presence of hHSF1 mutants, cells were infected with Ad-sh-hHSF1-KD1 (1 × 10^7^ p.f.u. per ml) for 2 h and maintained in normal medium for 22 h. They were then infected with each adenovirus expressing Flag-tagged hHSF1 mutants (1 to 5 × 10^6^ p.f.u. per ml) for 2 h and maintained with normal medium for a further 46 h. After being heat-shocked at 42 °C for 1 h, they were fixed with 4% paraformaldehyde in medium and treated as described above. To detect Flag-tagged hHSF1 mutants, the cells were incubated with anti-FLAG mouse IgG (1:200 dilution; M2, Sigma) in 2% milk/PBS at 4 °C overnight, and then with Alexa Fluor 568-conjugated goat anti-mouse IgG (Molecular Probes).

### Subcellular fractionation

Subcellular fractionation was carried out essentially as described previously^69^. Briefly, HeLa cells treated as described above were harvested, washed twice in ice-cold PBS and resuspended in homogenizing buffer (20 mM HEPES-KOH, pH 7.5, 10 mM KCl, 1.5 mM MgCl_2_, 1 mM sodium EDTA, 1 mM sodium EGTA and 1 mM DTT) containing 250 mM sucrose and protease inhibitors (1 μg ml^−1^ leupeptin, 1 μg ml^−1^ pepstatin A, and 1 mM phenylmethylsulfonyl fluoride). After incubation for 30 min on ice, the cells were homogenized using a tight fitting Dounce homogenizer (Weaton type A, 30 strokes), and centrifuged at 500 *g* for 5 min at 4 °C. The pellet was homogenized in buffer C (see Supplementary Methods) and centrifuged at 20,000 *g* for 30 min to obtain the nuclear fraction. On the other hand, the supernatant was centrifuged at 10,000 *g* for 20 min at 4 °C. The resulting supernatant was centrifuged at 100,000 *g* for 1 h at 4 °C to obtain the cytosolic fraction, and the pellet was washed three times with homogenizing buffer and solubilized in TNC buffer (10 mM Tris-acetate, pH 8.0, 0.5% NP40, and 5 mM CaCl_2_) containing protease inhibitors to obtain the mitochondrial fraction. Equivalent volumes of the fractions (fractions isolated from 2 to 10 × 10^5^ cells) were subjected to western blotting using rabbit antibody for SSBP1 (αmSSBP1-1) and HSP90 (αhHSP90c), or mouse IgG for SP1 (sc-420, Santa Cruz) or TOM20 (sc-17764, Santa Cruz).

### ChIP assay

ChIP assay was performed using a kit according to the manufacturer’s instructions (EMD Millipore)^24^, using antibodies for HSF1 (αmHSF1j, ABE1044, Millipore), SSBP1 (αmSSBP1-1), BRG1 (07-478, Millipore) and Pol II (CTD4H8, Millipore). PCR of enriched DNA was performed using primer sets at HSP70.3 promoter (a, −937 to −745; b, −760 to −538; c, −558 to −344; d, −356 to −154; e, −169 to +75; Supplementary Table 2). Real-time qPCR of ChIP-enriched DNAs was performed using primers for the dHSE, pHSE, pausing, coding and intergenic regions (Supplementary Table 3)^24^. Percentage input was determined by comparing the cycle threshold value of each sample to a standard curve generated from a five-point serial dilution of genomic input, and compensated by values obtained using normal IgG. IgG-negative control immunoprecipitations for all sites yielded <0.05% input. All reactions were performed in triplicate with samples derived from three experiments.

### Measurement of membrane potential and PTP opening

MEF cells, treated with heat shock at 42 °C for 1 h or a proteasome inhibitor MG132 for 6 h, were stained for 30 min at 37 °C in the dark with a fluorescent dye, TMRM (red), which accumulates in the mitochondria in a membrane potential-dependent manner, and DAPI (blue), as described previously^70^. A protonophore, FCCP (10 μM), was added for 10 min as a negative control of TMRM fluorescence. Fluorescence images were taken using Axiovert 200 fluorescence microscope (Zeiss), and the TMRM fluorescence intensity (arbitrary units) from three independent experiments was quantified using ImageJ. Mitochondrial PTP opening was examined using MitoProbe Transition Pore Assay Kit (Life Technologies) according to the manufacturer’s instructions. HeLa cells, treated with or without heat shock at 42 °C for 60 min, were incubated at 37 °C for 30 min with a membrane-permeable dye, calcein AM, in the presence or absence of CoCl_2_, which quenched the fluorescence from cytosolic calcein but not from mitochondrial calcein. An ionophore, ionomycin, was co-incubated as a negative control. The intensity of calcein fluorescence (arbitrary units) from three independent experiments was quantified using ImageJ.

### Statistical analysis

Data were analysed with Student’s *t*-test or analysis of variance. Asterisks in figures indicate significant differences (*P*<0.05 or 0.01). Error bars represent the standard deviations (s.d.) for more than three independent experiments.

## Additional information

The microarray data of heat shock-induced changes in the mRNA profiles of control, HSF1- or SSBP1-knockdown MEF cells have been deposited in the Gene Expression Omnibus under the accession number GSE61456.

**How to cite this article:** Tan, K. *et al.* Mitochondrial SSBP1 protects cells from proteotoxic stresses by potentiating stress-induced HSF1 transcriptional activity. *Nat. Commun.* 6:6580 doi: 10.1038/ncomms7580 (2015).

## Supplementary Material

Supplementary Information Supplementary Figures 1-8, Supplementary Tables 1-4, Supplementary Methods and Supplementary References
