# Supplementary material for: Mitochondrial SSBP1 protects cells from proteotoxic stresses by potentiating stress-induced HSF1 transcriptional activity
Source: Nat Commun. 2015 Mar 12;6:6580. doi: 10.1038/ncomms7580 (PMC4558571; doi:10.1038/ncomms7580)
Supplement: Supplementary Information — Supplementary Figures 1-8, Supplementary Tables 1-4, Supplementary Methods and Supplementary References [file ncomms7580-s1.pdf]

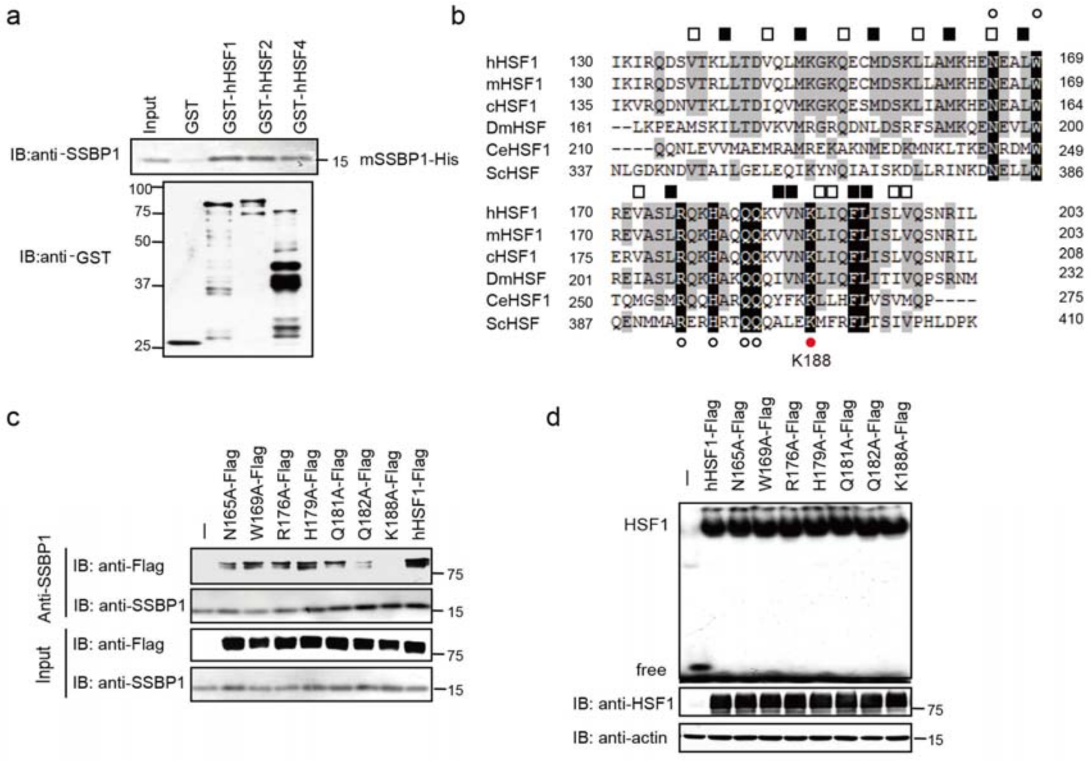

Supplementary Fig. 1. SSBP1 interacts with HSF1.

(**a**) mSSBP1 interacts with hHSF2 and hHSF4 as well as hHSF1. GST pull-down from mixtures of purified GST, GST-hHSF1, GST-hHSF2, or GST-hHSF4 with purified mSSBP1-His was performed, and blotted with SSBP1 or GST antibody. (**b**) Alignment of the amino acid sequences for the trimerization (HR-A/B) domain of human, mouse, and chicken HSF1, and *Drosophila melanogaster*, *C. elegans*, and *Saccharomyces cerevisiae* HSF. Residues identical among six sequences are indicated in black, and those identical among four or five are in gray. Open and solid squares show the heptad repeats of hydrophobic amino acids. Seven residues in hHSF1 that were identical to those in hHSF2, hHSF4 (see Fig. 1c), and HSF1 (or HSF) in other species are indicated by dots. Lysine at amino acid 188 (K188) (red dot) was required for the interaction with SSBP1. (**c**) Interaction of SSBP1 with HSF1 point mutants. Each HSF1 point mutant tagged with Flag at the C-terminus was overexpressed in HEK293 cells, and complexes co-immunoprecipitated using SSBP1 antibody were blotted with Flag or SSBP1 antibody. (**d**) DNA-binding activity of HSF1 point mutants. Whole cell extracts were prepared from cells treated as described in c, and were subjected to EMSA using ^{32}P-labeled HSE-oligonucleotide (upper). Western blotting was also performed (lower). Endogenous HSF1 was detected at a low level (lane 1).

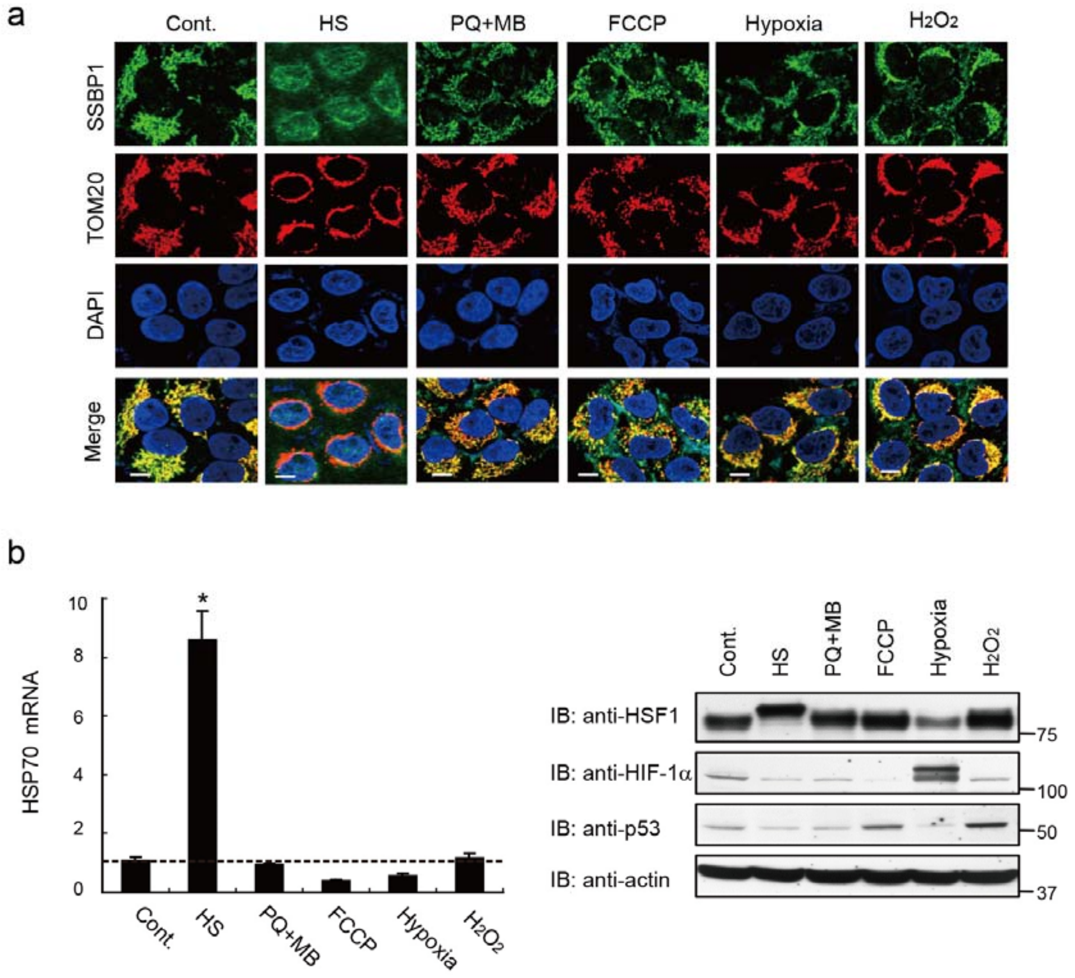

Supplementary Fig. 2. Localization of SSBP1 in response to various stresses.

(**a**) HeLa cells were treated without (Cont.) or with heat shock at 42°C for 60 min (HS), paraquat (PQ, 30 μM) and maneb (MB, 5 μM) for 6 h, FCCP (10 μM) for 6 h, hypoxia (1% O_2) for 16 h, H_2O_2 (0.4 mM) for 6 h. They were co-stained with antibodies for SSBP1 and TOM20, and DAPI. Each fluorescence image of SSBP1 (green), TOM20 (red), or DAPI (blue) in scanning confocal microscopy, and merged images were shown (Merge). Bars, 10 μm. (**b**) HeLa cells were treated as described in a. HSP70 mRNA levels were quantified by RT-qPCR, and the levels relative to those in untreated cells are shown (n=3) (left). Mean ± s.d. is shown. Asterisks indicate $p<0.05$ by Student's t-test. Western blotting was performed using antibodies for HSF1, HIF-1α (610958, BD Transduction Laboratories), p53 (sc-99, Santa Cruz), and β-actin.

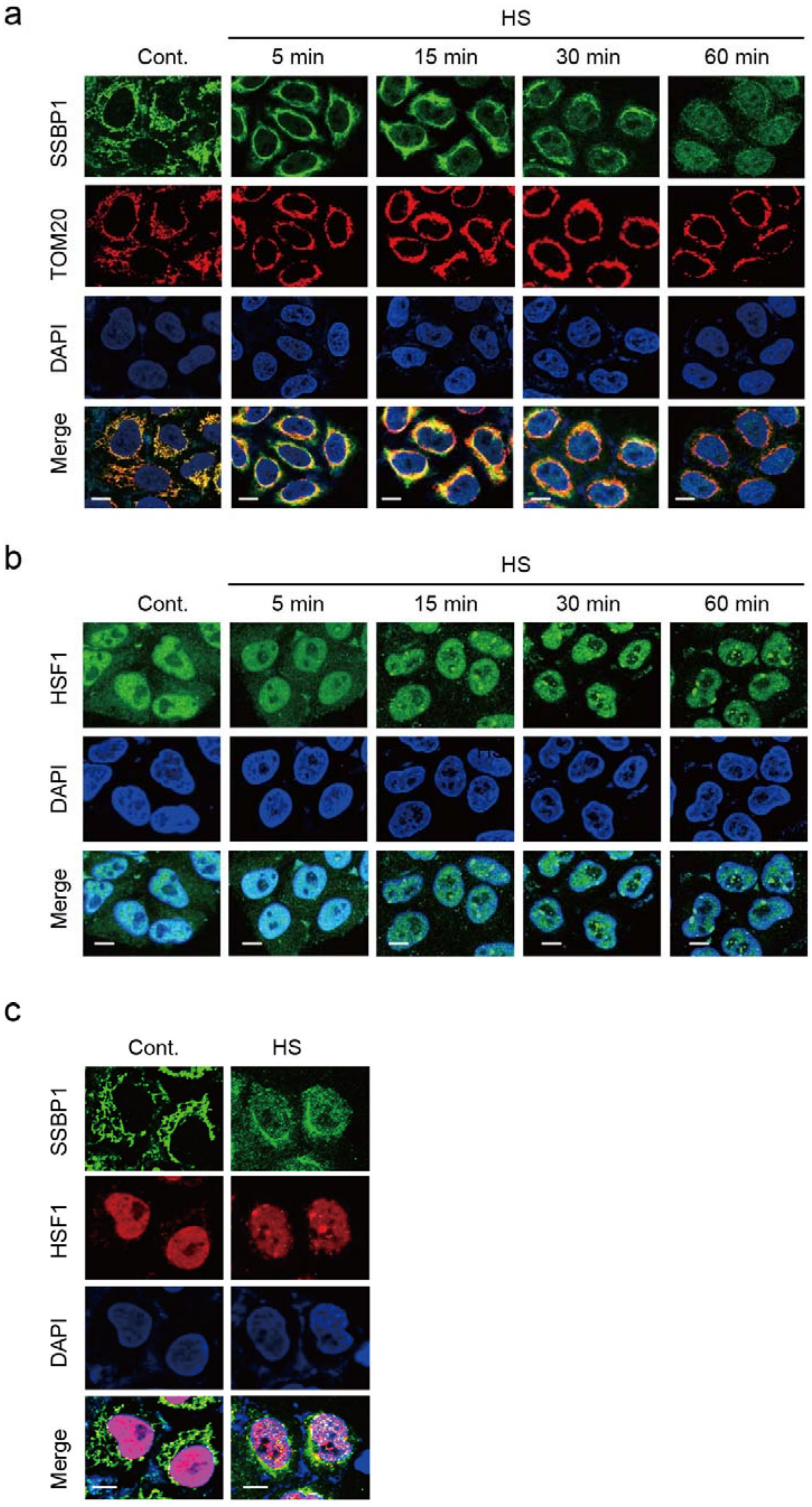
a
HS
Cont.
5 min
15 min
30 min
60 min
SSBP1
TOM20
DAPI
Merge
b
HS
Cont.
5 min
15 min
30 min
60 min
HSF1
DAPI
Merge
c
Cont.
HS
SSBP1
HSF1
DAPI
Merge

Supplementary Fig. 3. HSF1 and SSBP1 translocates to the nucleus during heat shock.
(**a**) HeLa cells were treated without (Cont.) or with heat shock at 42°C for 5, 15, 30, and 60 min (HS), and co-stained with antibodies for SSBP1 and TOM20, and DAPI. Each fluorescence image of SSBP1 (green), TOM20 (red), or DAPI (blue) in scanning confocal microscopy, and merged images were shown (Merge). Bars, 10 μm. (**b**) HeLa cells were treated as described in a, and co-stained with a polyclonal antibody for HSF1 (αcHSF1γ)[3] and DAPI. (**c**) HeLa cells were treated without (Cont.) or with heat shock at 42°C for 60 min (HS), and co-stained with antibodies for SSBP1 (green) and HSF1 (ab61382, Abcam) (red), and DAPI (blue). The HSF1 monoclonal antibody predominantly recognized the nuclear HSF1 in control condition. SSBP1 co-localized with HSF1 in the nucleus of heat-shocked cells. However, it did not accumulate at any focus unlike HSF1, possibly because the amount of SSBP1 protein is too low to appear as foci[5].

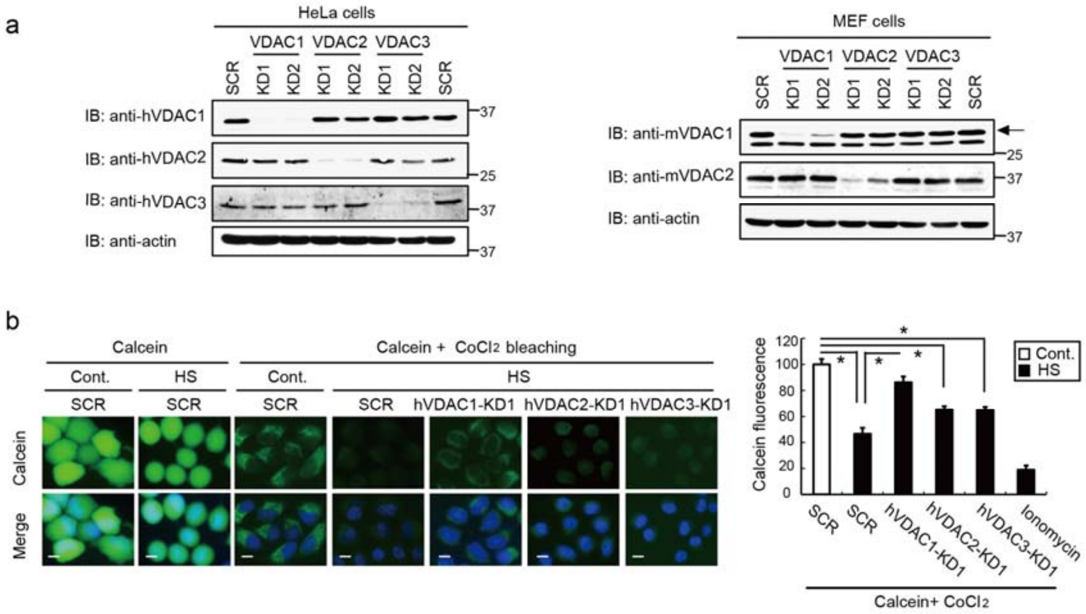

Supplementary Fig. 4. VDAC1 knockdown reduces mitochondrial PTP opening.

(**a**) Knockdown of VDAC members. HeLa (left) and MEF (right) cells were infected with Ad-sh-SCR or adenoviruses expressing shRNAs for VDAC1, VDAC2, or VDAC3 for 72 h. Cell extracts were prepared from these cells, and the expression of VDAC1, VDAC2, VDAC3, and β-actin were examined by Western blotting using antibodies for VDAC1 (ab14734, Abcam), VDAC2 (ab37985, Abcam), VDAC3 (sc-79341, Santa Cruz). An arrow indicates the position of mouse VDAC1. (**b**) Heat shock-mediated PTP opening is inhibited by VDAC1 knockdown. HeLa cells were infected with Ad-sh-SCR or adenovirus expressing shRNA for hVDAC1, hVDAC2, or hVDAC3 for 72 h, and then treated without (Cont.) or with heat shock at 42°C for 60 min (HS). These cells were incubated at 37°C for 30 min with calcein AM, in the presence or absence of $CoCl_2$. Ionomycin was co-incubated as a negative control. The intensity of calcein fluorescence (arbitrary units) from three independent experiments was quantified using ImageJ. Mean ± s.d. is shown. Asterisks indicate $p<0.05$ by Student's t-test.

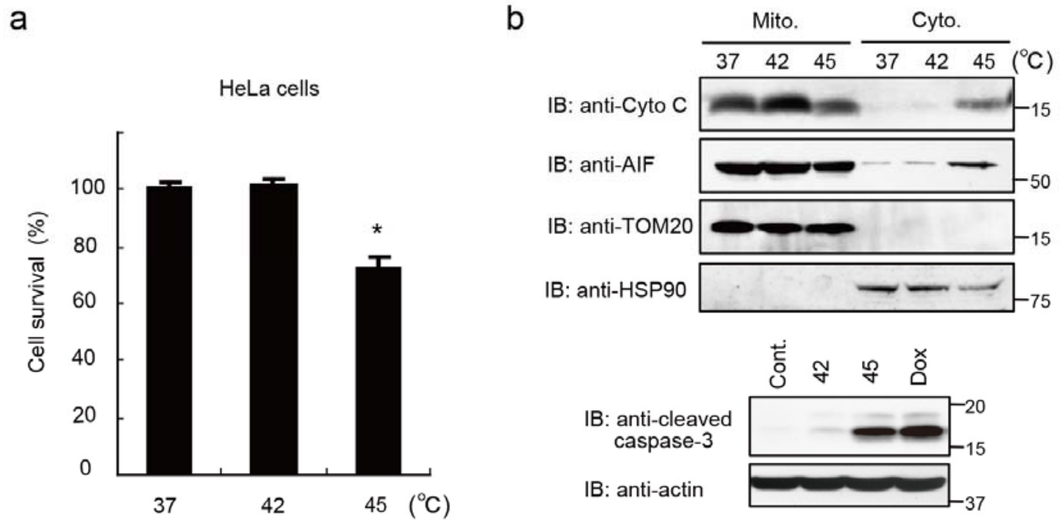

Supplementary Fig. 5. HeLa cells do not die during heat shock at 42°C for 1 h.

(**a**) Survival of HeLa cells during heat shock. Cells were treated without (37°C) or with moderate heat shock at 42°C for 1 h or extreme heat shock at 45°C for 2 h. The viable cells excluding trypan blue were counted and percentages of viable cells were shown (n=3). Mean ± s.d. is shown. Asterisks indicate $p<0.05$ by Student's t-test. (**b**) Release of apoptotic factors and cleavage of caspase-3. HeLa cells were treated as described in a. The mitochondrial (Mito.) and cytoplasmic (Cyto.) fractions were prepared and subjected to Western blotting using antibodies for Cyto C (7H8.2C12, BD Pharmingen), AIF (sc-13116, Santa Cruz), TOM20, and HSP90. Cells were treated with heat shock as described in a, and with 2 μM doxorubicin for 24 h (Dox). Total cell extracts were prepared and subjected to Western blotting using antibodies for cleaved caspase-3 (#9661, Cell Signaling) and β-actin.

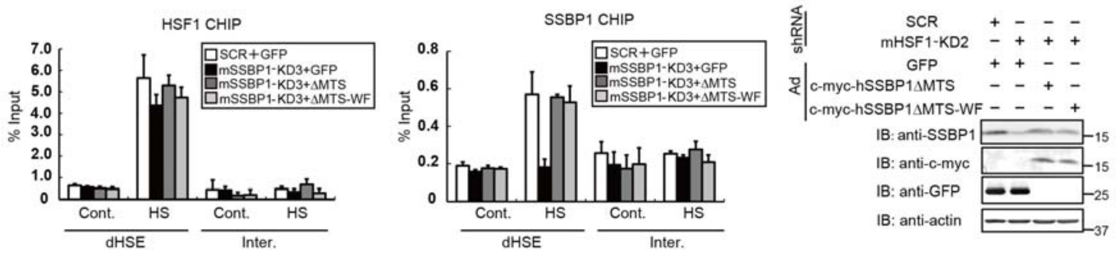

Supplementary Fig. 6. ssDNA-binding activity of SSBP1 is not required for its occupancy on HSF70.3 promoter.

Endogenous SSBP1 was substituted with c-myc-tagged hSSBP1ΔMTS (ΔMTS) or hSSBP1ΔMTS-W68T/F74A (ΔMTS-WF) that could not bind to a single-stranded DNA. These cells were untreated (Cont.) or treated with heat shock at 42°C for 30 min (HS). ChIP-qPCR analyses in the dHSE and an intergenic region (Inter.) were performed using HSF1 or SSBP1 antibody (n = 3) (left, middle). Mean ± s.d. is shown. Protein levels of SSBP1 and its mutants were examined by Western blotting using SSBP1 antibody or c-myc antibody (right).

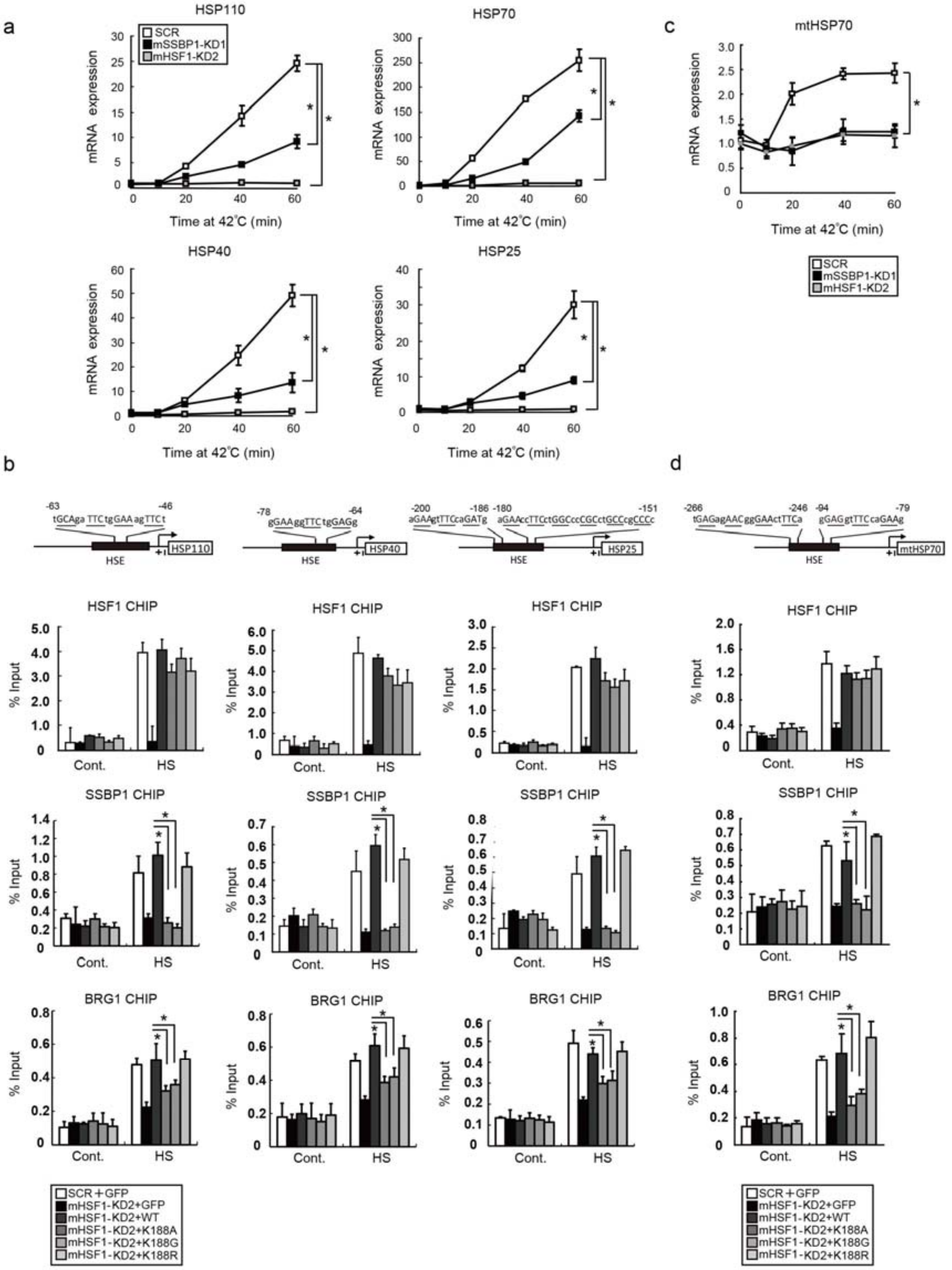
a
HSP110
HSP70
HSP40
HSP25
SCR
mSSBP1-KD1
mHSF1-KD2
mRNA expression
Time at 42°C (min)
c
mtHSP70
b
HSE
HSP110
HSP40
HSP25
d
mtHSP70
HSF1 CHIP
SSBP1 CHIP
BRG1 CHIP
% Input
Cont.
HS
SCR＋GFP
mHSF1-KD2+GFP
mHSF1-KD2+WT
mHSF1-KD2+K188A
mHSF1-KD2+K188G
mHSF1-KD2+K188R

Supplementary Fig. 7. HSF1-SSBP1 complex enhances the expression of cytoplasmic/nuclear and mitochondrial chaperones.

(**a**) Expression of cytoplasmic/nuclear chaperones during heat shock in SSBP1 or HSF1 knockdown cells. Cells were infected with Ad-sh-SCR, Ad-sh-mSSBP1-KD1, or Ad-sh-mHSF1-KD2 for 72 h, and heat-shocked at 42°C for the indicated periods. mRNAs for HSP110, HSP70, HSP40, and HSP25 were quantified by RT-qPCR, and levels relative to those in untreated cells are shown (n=3). Mean ± s.d. is shown. Asterisks indicate $p<0.05$ by ANOVA. (**b**) HSF1-SSBP1 complex promotes the recruitment of BRG1. Cells, in which endogenous HSF1 was replaced with each interaction mutant, were untreated (Cont.) or treated with heat shock at 42°C for 30 min (HS). ChIP-qPCR analyses of the each promoter were performed using antibody for HSF1, SSBP1, or BRG1 (n = 3). Mean ± s.d. is shown. Asterisks indicate $p<0.05$ by Student's t-test. (**c**) Expression of a mitochondrial chaperone, mtHSP70, during heat shock in SSBP1 or HSF1 knockdown cells. Cells were treated as described in a. mRNA for mtHSP70 was quantified by RT-qPCR, and relative levels are shown (n=3). (**d**) HSF1-SSBP1 complex promotes the recruitment of BRG1 to mtHSP70 promoter. Cells were treated as described in b and ChIP-qPCR analyses of the mtHSP70 promoter were performed.

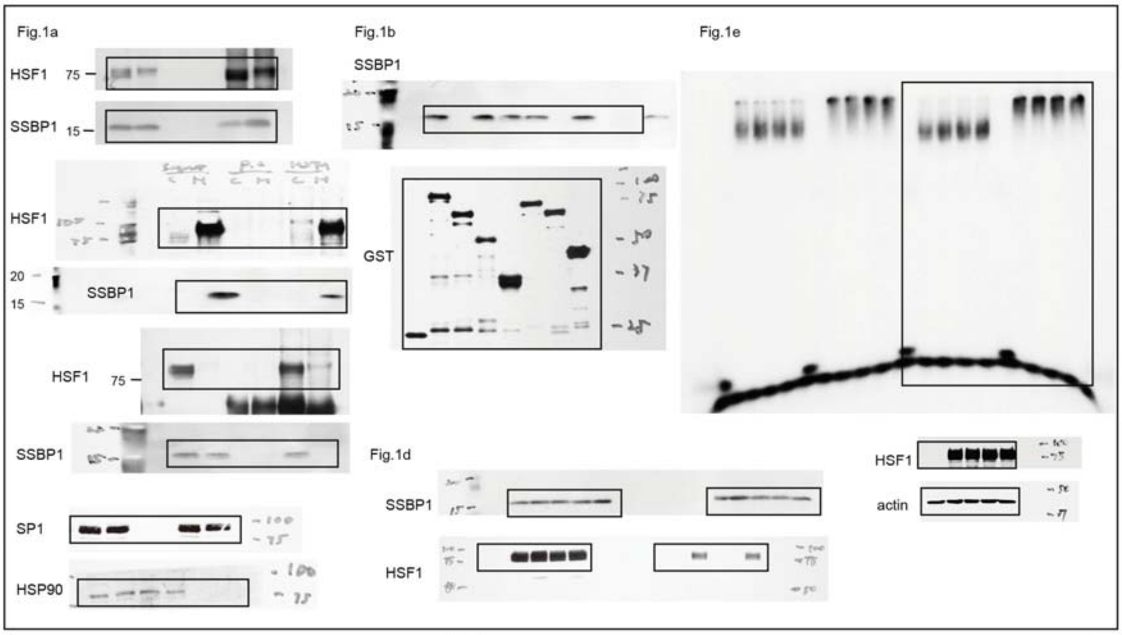
Fig.1a
HSF1 75
SSBP1 15
HSF1
20
15
SSBP1
HSF1 75
SSBP1
SP1
HSP90
Fig.1b
SSBP1
GST
Fig.1d
SSBP1
HSF1
Fig.1e
HSF1
actin

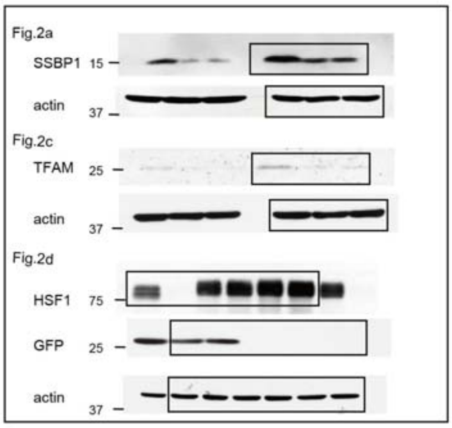
Fig.2a
SSBP1 15
actin 37
Fig.2c
TFAM 25
actin 37
Fig.2d
HSF1 75
GFP 25
actin 37

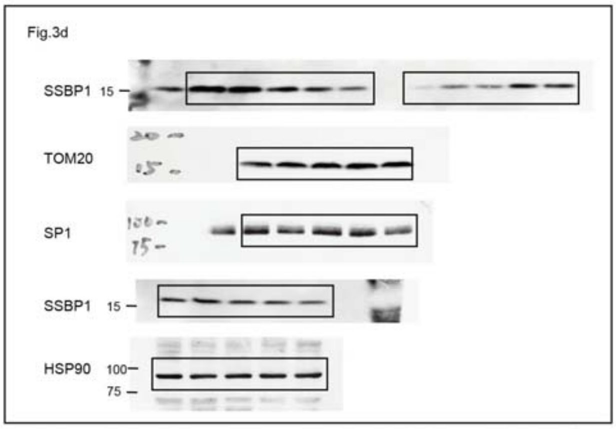
Fig.3d
SSBP1 15
TOM20
SP1
SSBP1 15
HSP90 100
75

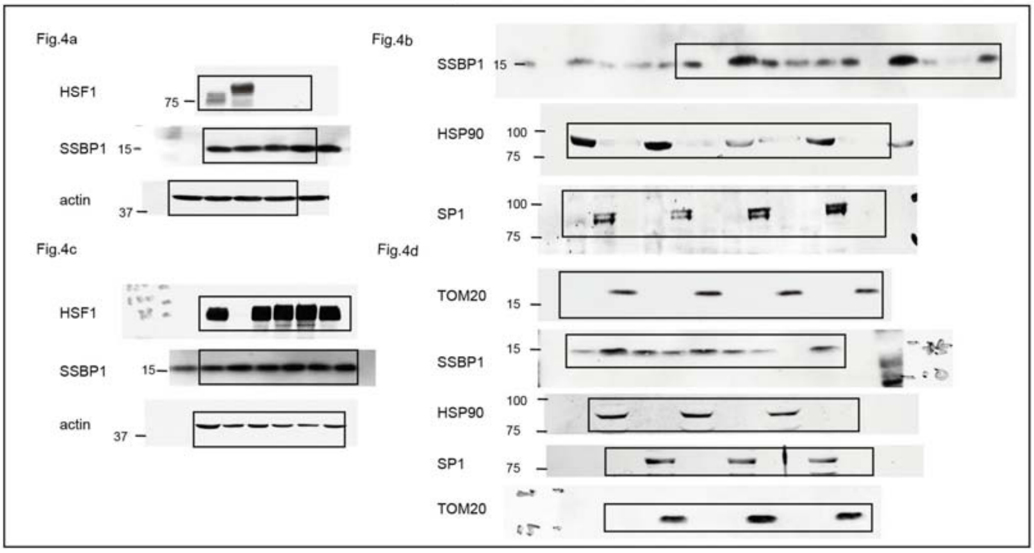
Fig.4a
HSF1 75
SSBP1 15
actin 37
Fig.4c
HSF1
SSBP1 15
actin 37
Fig.4b
SSBP1 15
HSP90 100
75
SP1 100
75
Fig.4d
TOM20 15
SSBP1 15
HSP90 100
75
SP1 75
TOM20

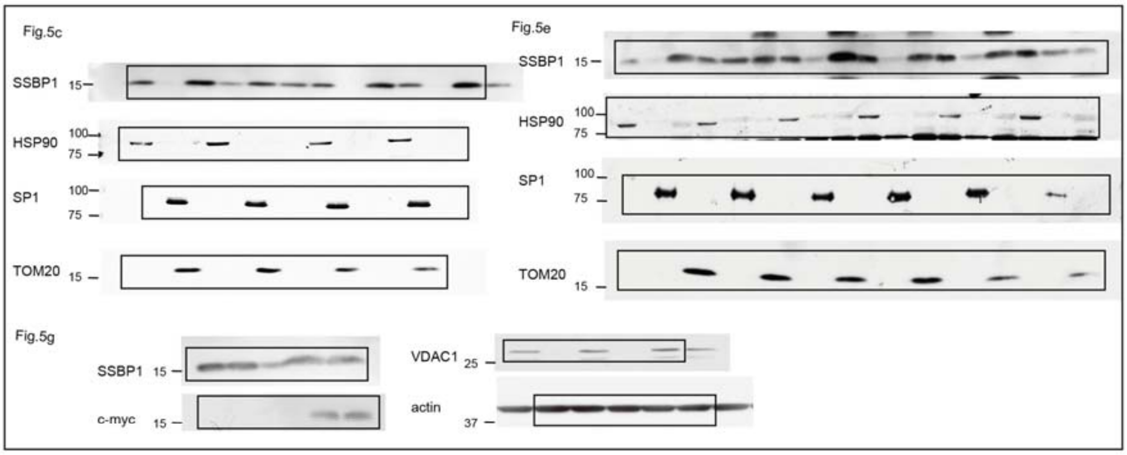
Fig.5c
SSBP1 15
HSP90 100 75
SP1 100 75
TOM20 15
Fig.5e
SSBP1 15
HSP90 100 75
SP1 100 75
TOM20 15
Fig.5g
SSBP1 15
c-myc 15
VDAC1 25
actin 37

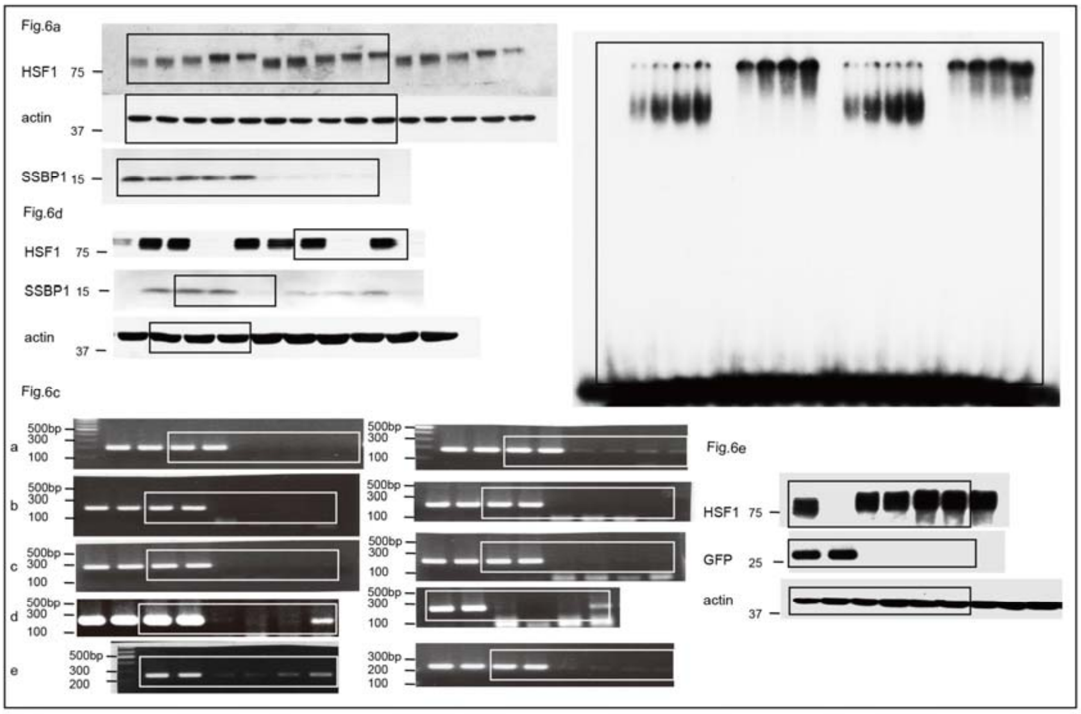
Fig.6a
HSF1 75
actin 37
SSBP1 15
Fig.6d
HSF1 75
SSBP1 15
actin 37
Fig.6c
a
b
c
d
e
500bp 300 100
500bp 300 200
300bp 200 100
Fig.6e
HSF1 75
GFP 25
actin 37

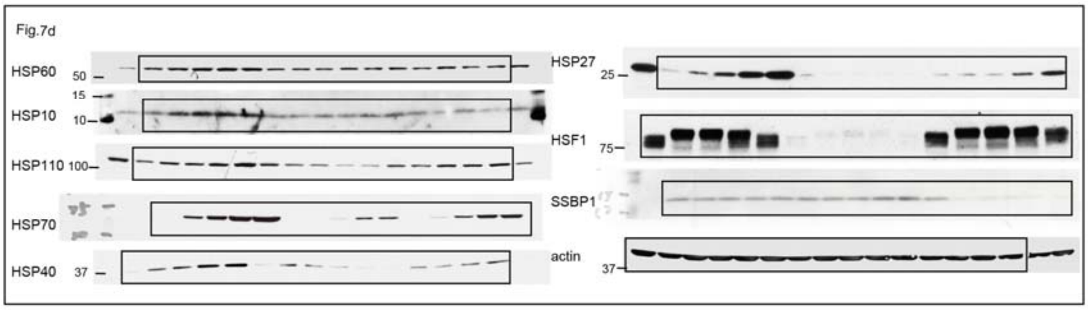
Fig.7d
HSP60 50
HSP10 15 10
HSP110 100
HSP70
HSP40 37
HSP27 25
HSF1 75
SSBP1
actin 37

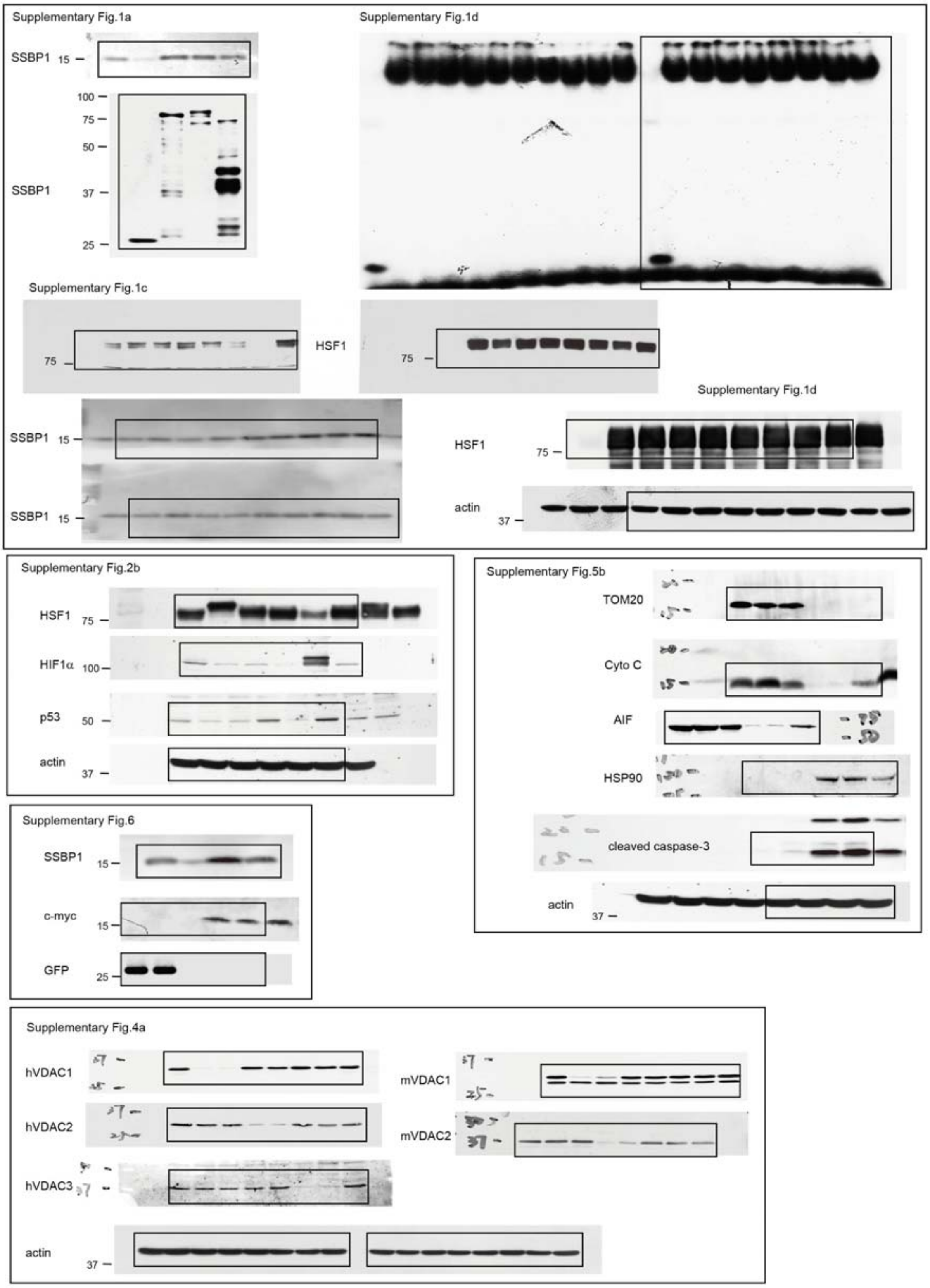

Supplementary Fig. 8. Uncropped scans of Western blots and gels of EMSA.

Supplementary Table 1. Primer sequences used for RT-qPCR

qPCR	Forward primer	Reverse primer
mHSP110	5'-GGATGCTGCGCAGATTGTG-3'	5'-GCAACAGCCGTCATGTCATT-3'
mHSP70	5'-GGCTGGTGAGCCACTTCGT-3'	5'-GTTCTGGCTGATGTCCTTCTTGT-3'
mHSP60	5'-GGCACTGGCTCCTCATCTCA-3'	5'-GCGTCCGCACCAAATTTT-3'
mHSP40	5'-CCTTCGGGAGGCTCTCTGT-3'	5'-GGTCCTGCCGTCCAGAGTAG-3'
mHSP25	5'-TCTCTCGGTGCTTCACCC-3'	5'-ATGGCTTCTACTTGGCTCCA-3'
mHSP10	5'-TGCTGCCGAAACTGTAACCA-3'	5'- TGCAACACTTTTCCTTGAGACTTT-3'
18S ribosomal RNA	5'-GTTCCGACCATAAACGATGCC-3'	5'-TGGTCGGTCCAGAAAAATCT-3'

Supplementary Table 2. Primer sequences used for ChIP-PCR

ChIP-PCR	Region	Forward primer	Reverse primer
mHSP70.3 promoter -937 to -745	a	5'-TTGATTGCCAACCACC AACCCCAAAAT-3'	5'-AAAGCCTCTGTGTCC CAGTTGGTCCTCGTT-3'
-760 to -538	b	5'-TGGGACACAGAGGCTT CTGCCCCACT-3'	5'-ATTTACCAACTAGAGG CTCTGTCCCAGC-3'
-558 to -344	c	5'-CAGAGCCTCTAGTTC CTAAATTAGTCCA-3'	5'-CAGTTTGTTGTGATTT GTGGGGTTTCG-3'
-356 to -154	d	5'-TCACAACAAACTGTAC ACAACACCGAG-3'	5'-CGGAGTTGTGGGTTCC GCCCTTGTCCA-3'
-169 to +75	e	5'-GGAACCCACAACTC CGATTA-3'	5'-CGCTCGCTCTGCT TCTCTT-3'

Supplementary Table 3. Primer sequences used for ChIP-qPCR

ChIP-qPCR	Forward primer	Reverse primer
mHSP110 promoter	5'-GAGGCGCCGGTGAGTAAA-3'	5'-CCTTATGTAGCCTACTGAGGAGAACTT-3'
mHSP40 promoter	5'-ATGTGCTGCGTCACGGAG-3'	5'-GCCAGCCCTCCAGAACCT-3'
mHSP60/HSP10 promoter	5'-GCCGAGGTGAAAGAACGA-3'	5'-TCCCGTGGGTGAAAGGT-3'
mHSP25 promoter	5'-TCGCTCCAGCTACCGGTATT-3'	5'-CCCCCATCTGGAAACTTCTCT-3'
mHSP70.3 dHSE	5'-ACCCTCCCCCTCAGGAATC-3'	5'-TGTCCAGAACTCTCCAGAGGTTT-3'
mHSP70.3 pHSE	5'-GATTACTCAAGGGAGGCGGG-3'	5'-TCCGCTGGGCCAATCA-3'
mHSP70.3 pausing	5'-TGACAGCTACTCAGAACCAAATCTG-3'	5'-TGGTCCTGGCCGAGGAT-3'
mHSP70.3 coding	5'-CAGCGAGGCTGACAAGAAGAA-3'	5'-CAGCTCCTCCCGCTTGTG-3'
mHSP70.3 intergenic	5'-GTGGCGCATGCCTTTGAT-3'	5'-CTTTGTAGAACAGGCTGACCTTGA-3'

Supplementary Table 4. Primer sequences used for gene knockdown

shRNA	Sense strand	Antisense strand
SCR	5'-GATCCATGTACTGCGCGTGGAGACTTCAAGA<br>GAGTCTCCACGCGCAGTACATTCTTTTGGAAA-3'	5'-AGCTTTTCCAAAAGAATGTACTGCGCGTGGA<br>GACTCTCTTGAAATCAGTCGTATTTCTCTTCG-3'
SSBP1<br>-KD1*	5'GATCCGACCTGTGTTACAGGTATTCTCAAGAGA<br>AATACCTGTAACACAGGTCTTTTTTGGAAA-3'	5'-AGCTTTTCCAAAAAAGACCTGTGTTACAGGTATTT<br>CTCTTGAGAATACCTGTAACACAGGTCG-3'
SSBP1<br>-KD2*	5'-GATCCGCAACAACAATCATAGCTGTTCAAGA<br>GACAGCTATGATTGTTGTTGCTTTTTTGGAAA-3'	5-'AGCTTTTCCAAAAAAGCAACAACAATCATAGCTG<br>TCTCTTGAACAGCTATGATTGTTGTTGCG-3'
SSBP1<br>-KD3*	5'-GATCCGCCTAAAGATTAGGTTGTATTCAAGA<br>GATACAACCTAATCTTTAGGCTTTTGGAAA-3'	5'-AGCTTTTCCAAAAAAGCCTAAAGATTAGGTTGTA<br>TCTCTTGAATACAACCTAATCTTTAGGCG-3'
VDAC1<br>-KD1*	5'-GATCCGTACAGATGGACTGAGTATTTCAAGA<br>GAATACTCAGTCCATCTGTACTTTTTTGGAAA-3'	5'-AGCTTTTCCAAAAAAGTACAGATGGACTGAGTAT<br>TCTCTTGAAATACTCAGTCCATCTGTACG-3'
VDAC1<br>-KD2*	5'-GATCCGAATGACGGGACAGAGTTTTTCAAGA<br>GAAAACTCTGTCCCGTCATTCACTTTTTTGGAAA-3'	5'-AGCTTTTCCAAAAAAGTGAATGACGGGACAGAG<br>TTTTCTCTTGAAAAACTCTGTCCCGTCATTCACTTCG-3'
VDAC2<br>-KD1*	5'-GATCCGTACAAATGGTGTGAGTATTTCAAGA<br>GAATACTCACACCATTTGTACTTTTTTGGAAA-3'	5'-AGCTTTTCCAAAAAAGTACAAATGGTGTGAGTAT<br>TCTCTTGAAATACTCACACCATTTGTACG-3'
VDAC2<br>-KD2*	5'-GATCCGCTGACAAGGAGTAACTTTTTCAAGA<br>GAAAAGTTACTCCTTGTCAGCTTTTTTGGAAA-3'	5'-AGCTTTTCCAAAAAAAGCTGACAAGGAGTAACTT<br>TTCTCTTGAAAAAGTTACTCCTTGTCAGCG-3'
VDAC3<br>-KD1*	5'-GATCCATCCAAACTGTCTCAGAATTTCAAGA<br>GAATTCTGAGACAGTTTGGATTTTTTGGAAA-3'	5'-AGCTTTTCCAAAAAAATCCAAACTGTCTCAGAAT<br>TCTCTTGAAATTCTGAGACAGTTTGGATG-3'
VDAC3<br>-KD2*	5'-GATCCGCAACCTAGAGACCAAATATTCAAGA<br>GATATTTGGTCTCTAGGTTGCCTTTTTTTGGAAA-3'	5'-AGCTTTTCCAAAAAAAGGCAACCTAGAGACCAAA<br>TATCTCTTGAATATTTGGTCTCTAGGTTGCG-3'
mTFAM<br>-KD1	5'-GATCCGATGAACCATTTGGTTAACTTCAAGA<br>GAGTTAACCAAATGGTTCATCTTTTTTGGAAA-3'	5'-AGCTTTTCCAAAAAAGATGAACCATTTGGTTAAC<br>TCTCTTGAAGTTAACCAAATGGTTCATCG-3'
mTFAM<br>-KD2	5'-GATCCGTGTAAAGAACATATTTCTTTCAAGA<br>GAAGAAATATGTTCTTTACACTTTTTTGGAAA-3'	5'-AGCTTTTCCAAAAAAGTGTAAAGAACATATTTCT<br>TCTCTTGAAAGAAATATGTTCTTTACACG-3'

*Sequences for both mouse and human genes.

Supplementary Methods

Plasmids

Adenovirus expressing shRNAs against mouse and human SSBP1, TFAM, VDAC1, VDAC2, VDAC3 were generated using oligonucleotides listed in Supplementary Table 4, as described previously[1]. To generate an expression vector, pmSSBP1-GFP or pmSSBP1ΔMTS-GFP, an XhoI/BamHI cDNA fragment of mSSBP1 or mSSBP1ΔMTS was amplified by PCR, and was inserted into pEGFP-N1 vector (Clontech). We generated an expression vector for c-myc-tagged proteins (pShuttle-myc-N) by replacing the multiple cloning site of pShuttle-CMV vector (Stratagene) with a KpnI/EcoRV oligonucleotide fragment containing a KOZAK sequence, a c-myc-coding sequence, and a set of cloning sites including XhoI and EcoRI. An XhoI/EcoRI cDNA fragment of c-myc-tagged hSSBP1ΔMTS was created by RT-PCR using total RNA isolated from HeLa cells, and was inserted into the pShuttle-myc-N vector. Adenovirus expression vector pAd-c-myc-hSSBP1ΔMTS was generated in accordance with the manufacturer's instructions. A cDNA for a mutant hSSBP1ΔMTS-W68T/F74A that cannot bind to ssDNA[2], was created by PCR, and its adenovirus expression vector was generated as described above. The sequences were verified using 3500 Genetic Analyzer (Applied Biosystems).

Electrophoretic mobility shift assay

Whole cell extracts were prepared in buffer C (20 mM HEPES, pH7.9, 25% glycerol, 0.42 M NaCl, 1.5 mM $MgCl_2$, 0.2 mM EDTA, 0.5 mM PMSF, and 0.5 mM DTT) from HEK293 cells transfected with expression vectors for hHSF1 or an hHSF1 mutant, or HSF1-knockdown MEF cells infected with adenovirus expressing hHSF1 or an hHSF1 mutant. Aliquots of the extracts (10 μg) were subjected to EMSA using an ideal HSE-oligonucleotide in the presence of preimmune serum or HSF1 antibody (α-HSF1γ) as described previously[3]. HSF1 and β-actin protein levels were determined by Western blotting.

GST pull-down assay

Bacterial expression vectors for GST-hHSF1, GST-hHSF2, GST-hHSF4, and hHSF1 mutants fused to GST were described previously[4]. Recombinant GST fusion proteins were expressed in *Escherichia coli* by incubating with 0.2 mM isopropyl β-D-1- thiogalactopyranoside (IPTG) at 25°C for 6 h, and purified using Glutathione Sepharose 4B (GE Healthcare). We constructed bacterial expression vector pET21a-mSSBP1-His by inserting a BamHI/XhoI fragment, which

was created by RT-PCR, into pET21a vector (Novagen). Recombinant His-tagged protein was similarly expressed and purified by Ni Sepharose 6 Fast Flow (GE Healthcare). The purified GST and His-tagged fusion proteins (1 μg each) were mixed in NT buffer (50 mM Tris-HCl pH7.5, 150 mM NaCl, 1 mM EDTA, 0.5% NP40, 1 mM PMSF, 1 μg/ml leupeptin, 1 μg/ml pepstatin) containing 0.25% gelatin and 0.02% NaN_3 at 4°C for 1 h, and were then incubated with 20 μl Glutathione Sepharose 4B at 4°C for 1 h. After the beads were washed with NT buffer five times, the bound proteins were analyzed by Western blotting using anti-SSBP1 (αmSSBP1-1) and anti-GST (αGST-S) antibodies.

Microarray analysis

Immortalized MEF cells (stock #10) were infected with Ad-sh-mHSF1-KD2, Ad-sh-mSSBP1-KD1, or Ad-sh-SCR (1 x 10^8 pfu/ml) for 2 h, maintained in normal medium for 70 h, and treated with heat shock at 42°C for 1 h. Total RNA was prepared using RNeasy Mini Kit (Qiagen). Microarray analysis was performed using GeneChip System (Affymetrix), and fold-changes for each gene were evaluated by Gene Expression Analysis using the software Partek Genomics Suite 6.5 (Partek) as described previously[4].

Supplementary references

1. Fujimoto, M. *et al.* A novel mouse HSF3 has the potential to activate nonclassical heat-shock genes during heat shock. *Mol. Biol. Cell* **21,** 106-116 (2010).
2. Li, K. & Williams, R.S. Tetramerization and single-stranded DNA binding properties of native and mutated forms of murine mitochondrial single-stranded DNA-binding proteins. *J. Biol. Chem.* **272,** 8686-8694 (1997).
3. Shinkawa, T. *et al.* Heat shock factor 2 is required for maintaining proteostasis against febrile-range thermal stress and polyglutamine aggregation. *Mol. Biol. Cell* **22,** 3571-3583 (2011).
4. Fujimoto, M. *et al.* RPA assists HSF1 access to nucleosomal DNA by recruiting histone chaperone FACT. *Mol. Cell* **48,** 182-194 (2012).
5. Eymery, A., Souchier, C., Vourc'h, C. & Jolly, C. Heat shock factor 1 binds to and transcribes satellite II and III sequences at several pericentromeric regions in heat-shocked cells. *Exp. Cell Res.* **316,** 1845-1855 (2010).
